# MCL1 inhibitors S63845/MIK665 plus Navitoclax synergistically kill difficult-to-treat melanoma cells

**DOI:** 10.1038/s41419-020-2646-2

**Published:** 2020-06-08

**Authors:** Nabanita Mukherjee, Jenette Skees, Kaleb J. Todd, Drake A. West, Karoline A. Lambert, William A. Robinson, Carol M. Amato, Kasey L. Couts, Robert Van Gullick, Morgan MacBeth, Kelsey Nassar, Aik-Choon Tan, Zili Zhai, Mayumi Fujita, Stacey M. Bagby, Chiara R. Dart, James R. Lambert, David A. Norris, Yiqun G. Shellman

**Affiliations:** 1 Department of Dermatology, University of Colorado Anschutz Medical Campus, School of Medicine, Mail Stop 8127, Aurora, CO 80045 US; 2 University of Colorado Anschutz Medical Campus, School of Medicine, Division of Medical Oncology, Mail Stop 8117, Aurora, CO 80045 US; 3 Department of Biostatistics and Bioinformatics, Moffitt Cancer Center, 12902 Magnolia Drive, Tampa, FL 33612 US; 4 Department of Pathology, University of Colorado Anschutz Medical Campus, School of Medicine, Mail Stop 8104, Aurora, CO 80045 US; 5 Department of Veterans Affairs Medical Center, Dermatology Section, Denver, CO 80220 US; 6 University of Colorado Anschutz Medical Campus, Gates Center for Regenerative Medicine, Aurora, CO 80045 US

**Keywords:** Melanoma, Melanoma

## Abstract

Current treatment for patients with metastatic melanoma include molecular-targeted therapies and immune checkpoint inhibitors. However, a subset of melanomas are difficult-to-treat. These melanomas include those without the genetic markers for targeted therapy, non-responsive to immunotherapy, and those who have relapsed or exhausted their therapeutic options. Therefore, it is necessary to understand and explore other biological processes that may provide new therapeutic approaches. One of most appealing is targeting the apoptotic/anti-apoptotic system that is effective against leukemia. We used genetic knockdown and pharmacologic approaches of BH3 mimetics to target anti-apoptotic BCL2 family members and identified MCL1 and BCLXL as crucial pro-survival members in melanoma. We then examined the effects of combining BH3 mimetics to target MCL1 and BCLXL in vitro and in vivo. These include clinical-trial-ready compounds such as ABT-263 (Navitoclax) and S63845/S64315 (MIK655). We used cell lines derived from patients with difficult-to-treat melanomas. In vitro, the combined inhibition of MCL1 and BCLXL resulted in significantly effective cell killing compared to single-agent treatment (*p* < 0.05) in multiple assays, including sphere assays. The combination-induced cell death was independent of BIM, and NOXA. Recapitulated in our mouse xenograft model, the combination inhibited tumor growth, reduced sphere-forming capacity (*p* < 0.01 and 0.05, respectively), and had tolerable toxicity (*p* > 0.40). Taken together, this study suggests that dual targeting of MCL1 and BCLXL should be considered as a treatment option for difficult-to-treat melanoma patients.

## Introduction

The incidence of invasive melanoma cases has increased by 54% in the last decade^1^. Treatment of advanced melanoma has dramatically improved in recent years, and currently include targeted therapies against BRAF or MEK, and immunotherapy. Targeted therapies work only on a subset of patients with specific mutations; however, of patients that initially respond, almost all relapse. Although promising, immunotherapies are not without caveats—not all patients respond and some patients relapse^2–4^. Thus, it is important to find alternative melanoma treatment options, targeting biological processes that are different from current therapies.

Resistance to cell death is one hallmark of cancer, and the BCL2 family of proteins plays a crucial role in regulating this process. The upregulation of pro-survival/anti-apoptotic members contributes to tumorigenesis, and to resistance to both generalized chemotherapy and molecular-targeted therapies^5,6^; in short, these proteins over-ride the cell’s death triggers. The BCL2 family includes three groups based on their functions and structures: (1) multi-BH domain pro-apoptotic proteins (BAX and BAK) are effectors of apoptosis; (2) pro-survival proteins (BCL2, BCLXL, BCLW, MCL1, and BFL1) keep the effectors in check and inhibit cell death; and (3) BH3-only pro-apoptotic proteins (NOXA, BAD, BIM, tBID, and PUMA) are initiators of cell death that neutralize certain pro-survival proteins^5^. Interactions between different members are not mutually exclusive or equal—various combinations of interactions control the initiation of apoptosis.

BH3 mimetics represent a novel class of cancer therapeutic drugs, and act through a different mechanism than those currently used for the treatment of advanced melanoma. They are small molecule compounds that mimic the function of BH3 only proteins. They bypass upstream initiators of apoptosis such as p53 and act by binding pro-survival BCL2 family members, thereby activating the cell death pathway^7^. These mimetics have generated significant interest due to the remarkable efficacy of ABT-199 (venetoclax) in the treatment of hematological malignancies^8,9^.

Several BH3 mimetics targeting other pro-survival members are currently in clinical trials, including the pan BCL2 family inhibitor navitoclax (ABT-263) and MCL1 inhibitors S63845/S64315 (MIK665) (clinical trials.gov; NCT03672695; NCT01989585). The clinical effectiveness of targeting the apoptotic pathway in leukemia led us to examine its utility in melanoma, and we explored the therapeutic potential of these newer BH3 mimetics in vitro and in vivo. Our genetic knockdown of several BCL2 family members in combination with BH3 mimetics highlights the role of MCL1 and BCLXL in melanoma cells.

This study explored the therapeutic potential and mechanisms-of-action of the newest generation of BH3 mimetics in melanoma, using genetic (shRNA or CRISPR/Cas9 technology) and small molecule (BH3 mimetics) approaches, in vitro and in vivo. We used difficult-to-treat melanoma cell lines established from melanoma patients, with diverse tumor genetic backgrounds and rare types of melanomas (Supplementary Table 1). We demonstrate that combinations of drugs targeting both of these proteins, including compounds already in clinical trials for other cancers, have significant antitumor activity in human melanoma cell lines and in vivo murine xenografts. These data provide strong support for advancing these combinations into clinical trials.

## Results

### Knockdown of BCLXL sensitizes melanoma to MCL1 inhibitor, while knockdown of MCL1 sensitizes melanoma to BCLXL inhibitors

**Fig. 1:**
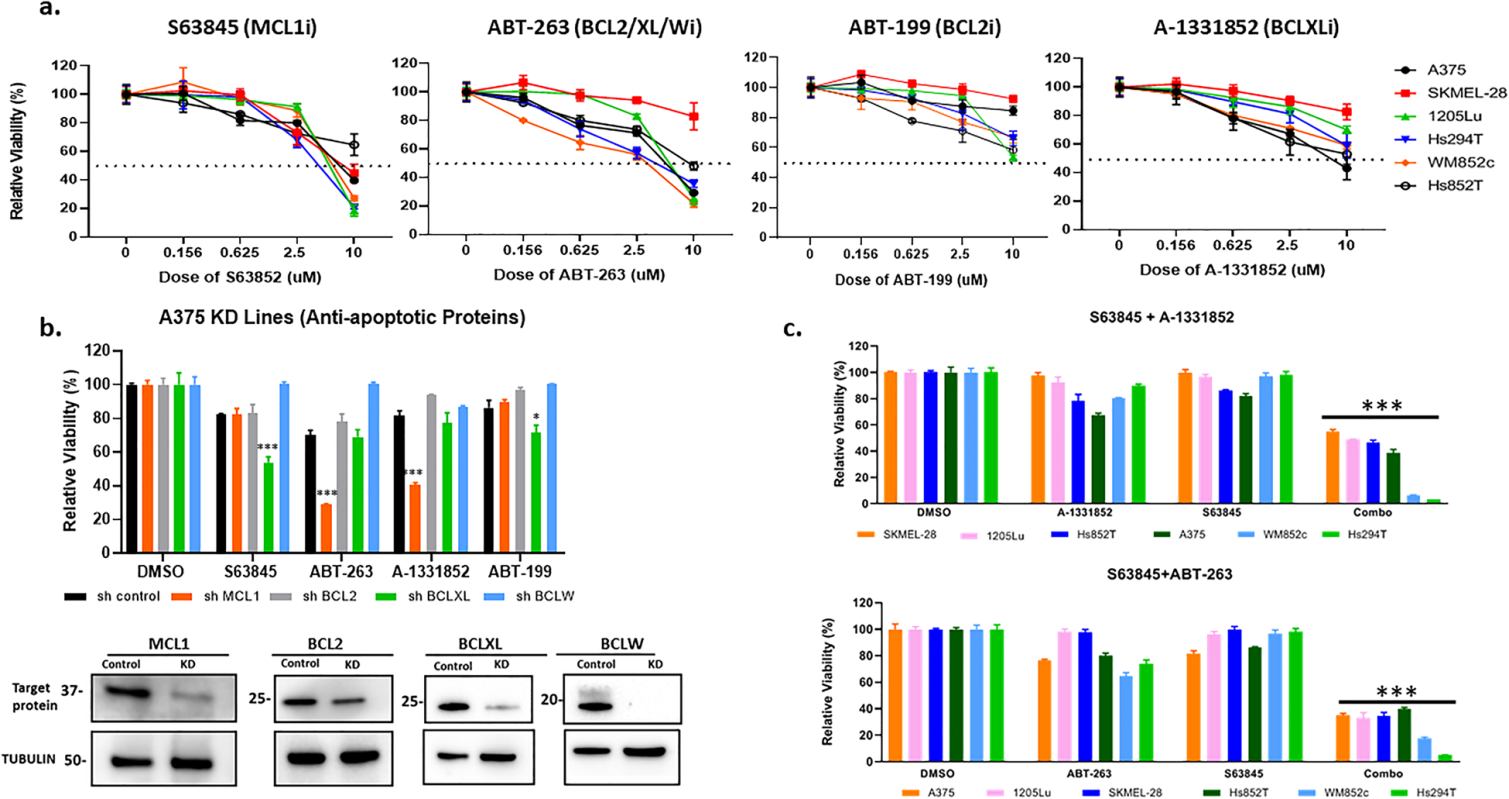
Single-drug treatment of BH3 mimetics had little effect on; combination treatment of MCL1 and BCLXL synergistically killed melanoma cells. **a** ATP assay of BH3 mimetic single-drug treatments of S63845 (MCL1 inhibitor), ABT-263 (BCL2/XL/W inhibitor) and A-1331852 (BCLXL inhibitor) on melanoma cell lines. Error bars represent ± SEM. *Y*-axis shows percentage of relative viability (to DMSO) and *X*-axis indicates the dosages of drug in µM. **b** In A375 cells, knockdown (KD) of MCL1 (shMCL1), BCL2 (shBCL2), BCLW (shBCLW), and BCLXL (shBCLXL) lines were created by shRNA technology. Only shMCL1 (in presence of ABT-263 or A-1331852) and shBCLXL (in presence of S63845) showed significant reduction in cell viability during 48 h drug treatment. *Y*-axis shows percentage of relative viability and *X*-axis indicates the BH3 mimetics used. Inset showing the immunoblots confirming the KD. Molecular weight markers are in kDa. **c** Summary of ATP assay data of ten melanoma cell lines treated with S63845 + A-1331852 or S63845 + ABT-263. For **c** all drugs were used at a dose of 156 nM. For visual clarity, we marked only the combinational treatments that were significantly different from comparisons with the DMSO and the single-drug treatments. Within each significant combination treatment, we only show the most significant *p*-value of the comparisons. *Indicates *p* < 0.05; ***indicates *p* < 0.001. Error bars represent ± SEM.

We first tested the effects of the newest generation of BH3 mimetics: ABT-199 (BCL2 inhibitor, Venetoclax), S63845 (MCL1 inhibitor), A-1331852 (BCLXL inhibitor), or ABT-263 (BCL2/BCLXL/BCLW inhibitor, Navitoclax) as single drugs (Fig. 1a; Supplementary Table 2). There was no sensitivity as single drug at less than 2 μM.

We and others have previously shown that most melanoma cell lines express the major anti-apoptotic BCL2 family member proteins, such as BCL2, MCL1, and BCLXL^10–15^. However, there has been no thorough mechanistic studies in melanoma that determines which specific BCL2 family members are crucial for resistance to single agent BH3 mimetic treatment. To determine if silencing these proteins would sensitize the cells to treatment with BH3 mimics, we used knockdown with shRNAs, followed by treatment with BH3 mimetics (Fig. 1b). Cell viability assays showed that MCL1 knockdown significantly sensitized cells to BCLXL inhibitors (ABT-263 or A-1331852), while BCLXL knockdown significantly sensitized cells to MCL1 inhibitor S63845 (*p* < 0.001) (Fig. 1b). Other knockdowns did not have significant effects on cell sensitivity. These results imply that targeting MCL1 and BCLXL in a combination treatment is an effective way to induce melanoma cell death.

We then tested the combinations of an MCL1 inhibitor (S63845) with either a BCLXL specific inhibitor (A-1331852) or a pan BCL2 inhibitor that also inhibits BCLXL (ABT-263). These combinations were very potent in reducing cell viability at sub-micromolar doses in a majority of melanoma cell lines (Fig. 1c, and Supplementary Figs. 1 and 2). Interestingly, these combinations had similar effects in most cases, indicating that MCL1 and BCLXL are essential BCL2 family members for melanoma survival.

### Combinational treatments targeting both MCL1 and BCLXL synergistically kill melanoma patient derived cell lines of diverse genetic backgrounds and melanoma subtypes

To determine whether the findings above are relevant to the current treatment of melanoma, we evaluated the efficacy of MCL1 plus BCLXL inhibition in a panel of patient derived lines with diverse genetic backgrounds established from several subtypes of melanomas (Supplementary Table 1), with the same conditions as in Fig. 1c and Supplementary Fig. 1. For example, the samples included cells with mutations in *BRAF*^*V600E*^ (MB2114), *BRAF* Fusion (MB1692), *NRAS* (MB3961, and MB3616), or were triple-WT (wild type for *BRAF*, *NRAS*, and *NF-1*; MB2724). The cell lines also included melanoma subtypes of superficial spreading, nodular, acral, and mucosal.

**Fig. 2:**
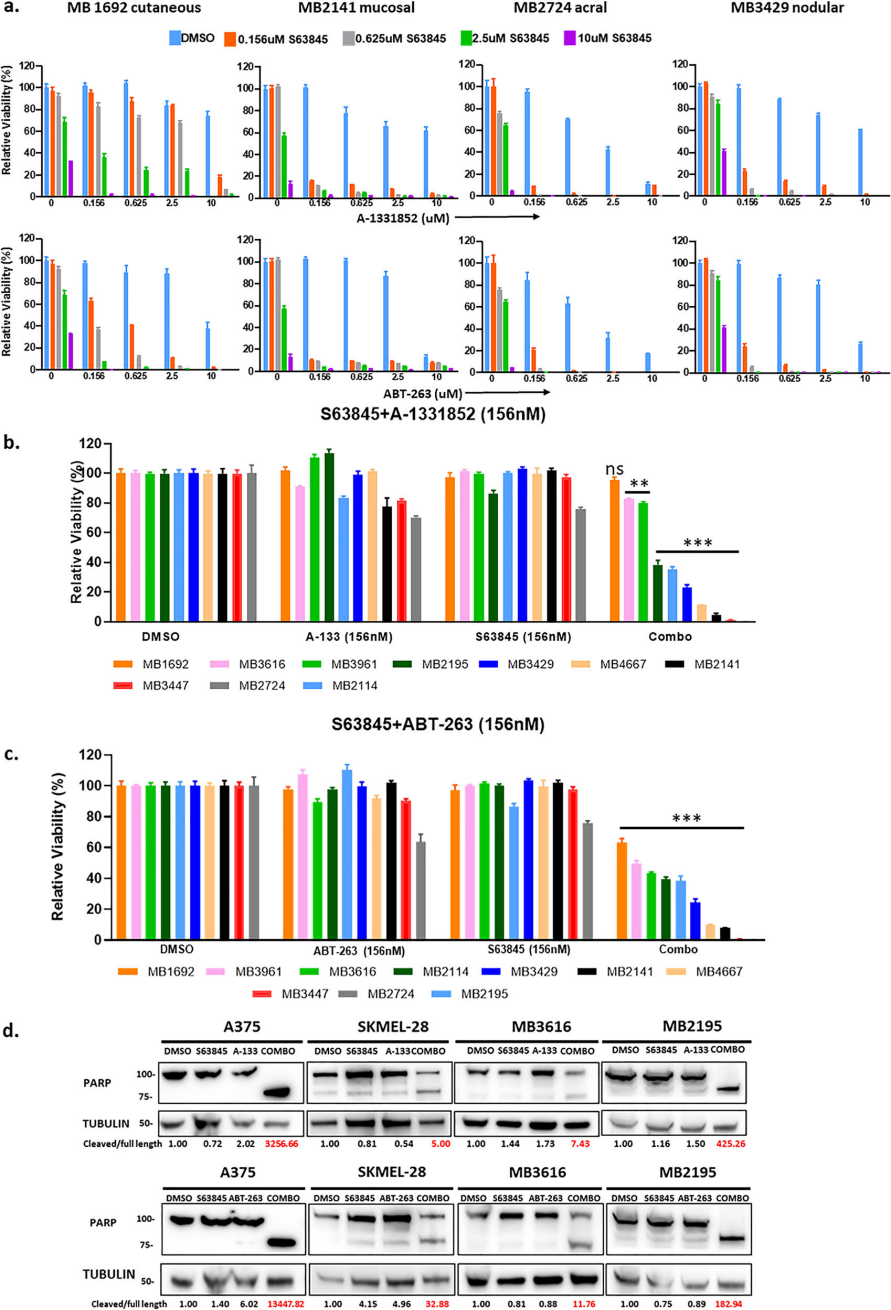
Combination therapy of BH3 mimetics (S63845+ABT-263 or S63845+A-1331852) synergistically killed melanoma samples of diverse genetic backgrounds. **a**, **b** ATP assays of four subtypes of melanoma patient samples upon indicated treatments for 48 h. The viability of the DMSO control for each cell line was set to 100%. Both the combinations (S63845+A-1331852 in (**a**); S63845+ABT-263 in (**b**)) significantly (*p* ≤ 0.01) reduced cell viability compared with DMSO or with single drug treated conditions in all melanoma cell lines at sub-micromolar doses. For visual clarity, the * is not shown in the figure. Both the combinations were highly synergistic at sub-micromolar doses (Supplementary Fig. 2). **c** Summary of ATP assay data of ten melanoma patient samples treated with S63845+A-1331852 or S63845+ABT-263. For **c** all drugs were used at a dose of 156 nM. For visual clarity, we marked only the combinational treatments that were significantly different from comparisons with the DMSO and the single-drug treatments. Within each significant combination treatment, we only show the most significant *p*-value of the comparisons. **Indicates *p* < 0.01; ***indicates *p* < 0.001. Error bars represent ± SEM. **d** Immunoblot with lysates collected after 48 h treatment with DMSO, single drugs, or combinations, and probed for PARP. Both combinations increased the cleaved product of PARP. Molecular weight markers are in kDa.

Both combinations had similar effects on the 10 patient lines (Fig. 2). Combination treatment significantly (*p* < 0.01) reduced cell viability compared to DMSO or single drug at nM doses (Fig. 2a, b and c). The effects were highly synergistic for the majority of conditions, with Combination Index (CI) values less than 0.5 (Supplementary Fig. 2). In contrast, the same nM doses of these drug treatments had only modest effects in primary melanocyte cells HEM_N_MP2 (Supplementary Fig. 3). Moreover, neither the subtype nor mutation status of BRAF or NRAS were predictive of sensitivity to combination treatments.

**Fig. 3:**
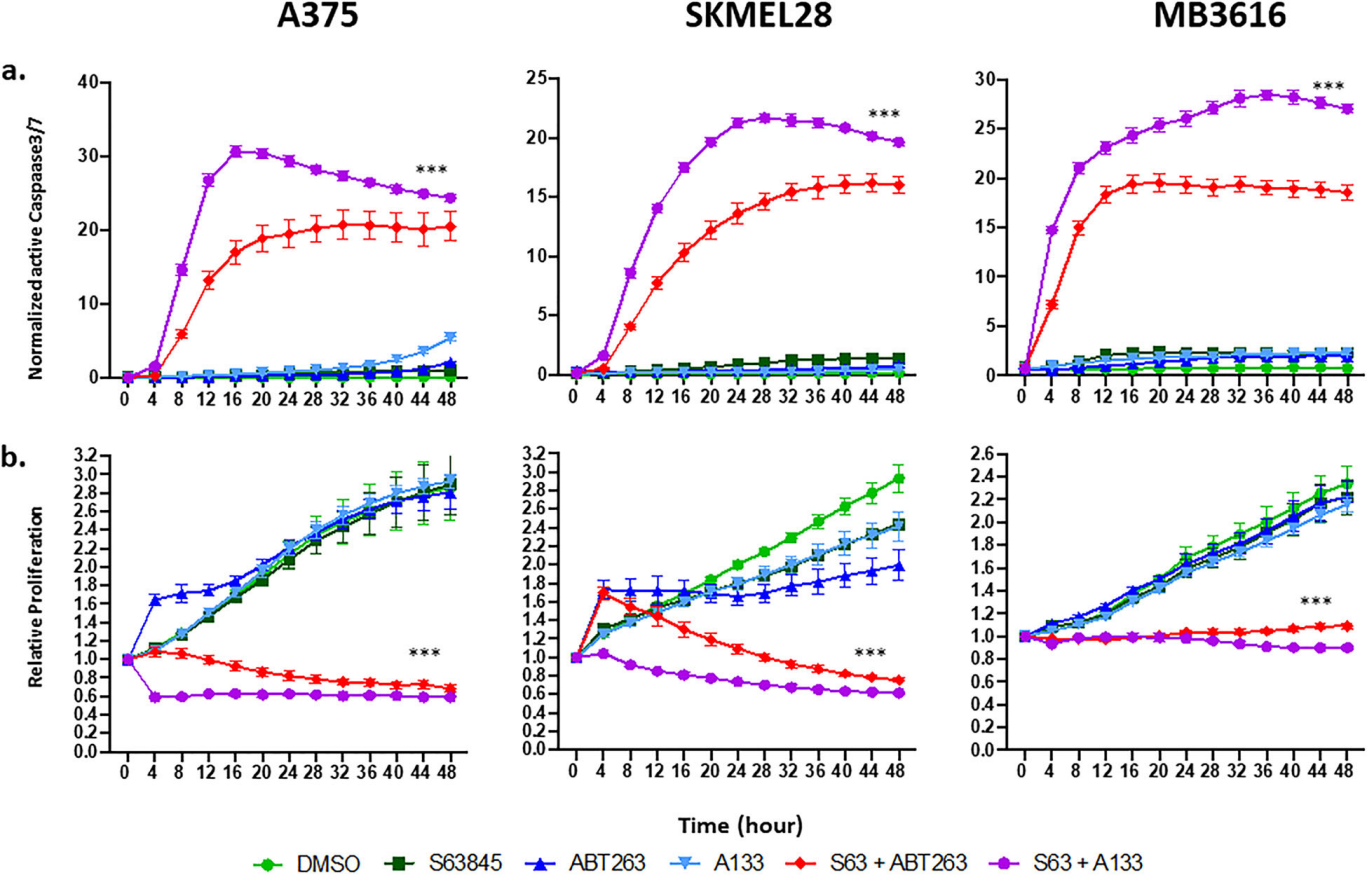
Combination treatments of BH3 mimetics (S63845 plus ABT-263 or A-1331852) induced apoptosis in melanoma cell lines and patient samples. IncuCyte live cell imaging with active Caspase 3/7 analyses to study apoptosis (**a**) and proliferation (**b**). Images were acquired using the phase and green fluorescent channels every 4 h for a total of 48 h. For visual clarity, we marked only the combinational treatments that were significantly different from comparisons with the DMSO and the single-drug treatments. Both the combination treatments significantly increased apoptosis and decreased proliferation compared to vehicle or single drugs. ***Indicates *p* < 0.001. Error bars represent ± SEM.

Immunoblots of cleaved PARP, a well-known marker of cellular apoptosis^16^, indicated that the combination treatment consistently induced more apoptosis relative to other treatments for all melanoma cell lines tested, irrespective of their BRAF or NRAS mutation status (Fig. 2d). Cell death was also visually verified by rounded morphology or complete cell detachment in combination treated plates (Supplementary Fig. 4). To further quantify the effects on apoptosis and proliferation, we performed IncuCyte live cell imaging analyses of both active Caspase 3/7 and confluency (Fig. 3, Supplementary Fig. 5 and Supplementary Video 1, 2, and 3). Both combination treatments S63845+A-1331852 (S63+A-133) and S63845+ABT-263 (S63+ABT-263) significantly increased Caspase 3/7 activation and decreased proliferation (*p* < 0.001), compared with vehicle or single-drug treatments (Fig. 3a, b). These data demonstrated that both combinations significantly induced apoptosis in multiple melanoma cell lines.

### Combined treatment against MCL1 and BCLXL killed the heterogeneous, resistant melanoma initiating cells (MICs) populations, and inhibited their self-renewability across multiple melanoma cell lines and patient samples

Cancer heterogeneity is a challenging issue while designing therapy. Like other cancers, melanoma is aggressive and therapy resistant due to its heterogeneity. Melanoma has a heterogeneous sub-population attributed with plasticity, stem-like features, and drug resistance, which may also contribute to relapse^17,18^. Thus, it is crucial to eliminate the heterogeneous population of cells to prevent relapse. We are using the term MICs to define the above-mentioned population.

**Fig. 4:**
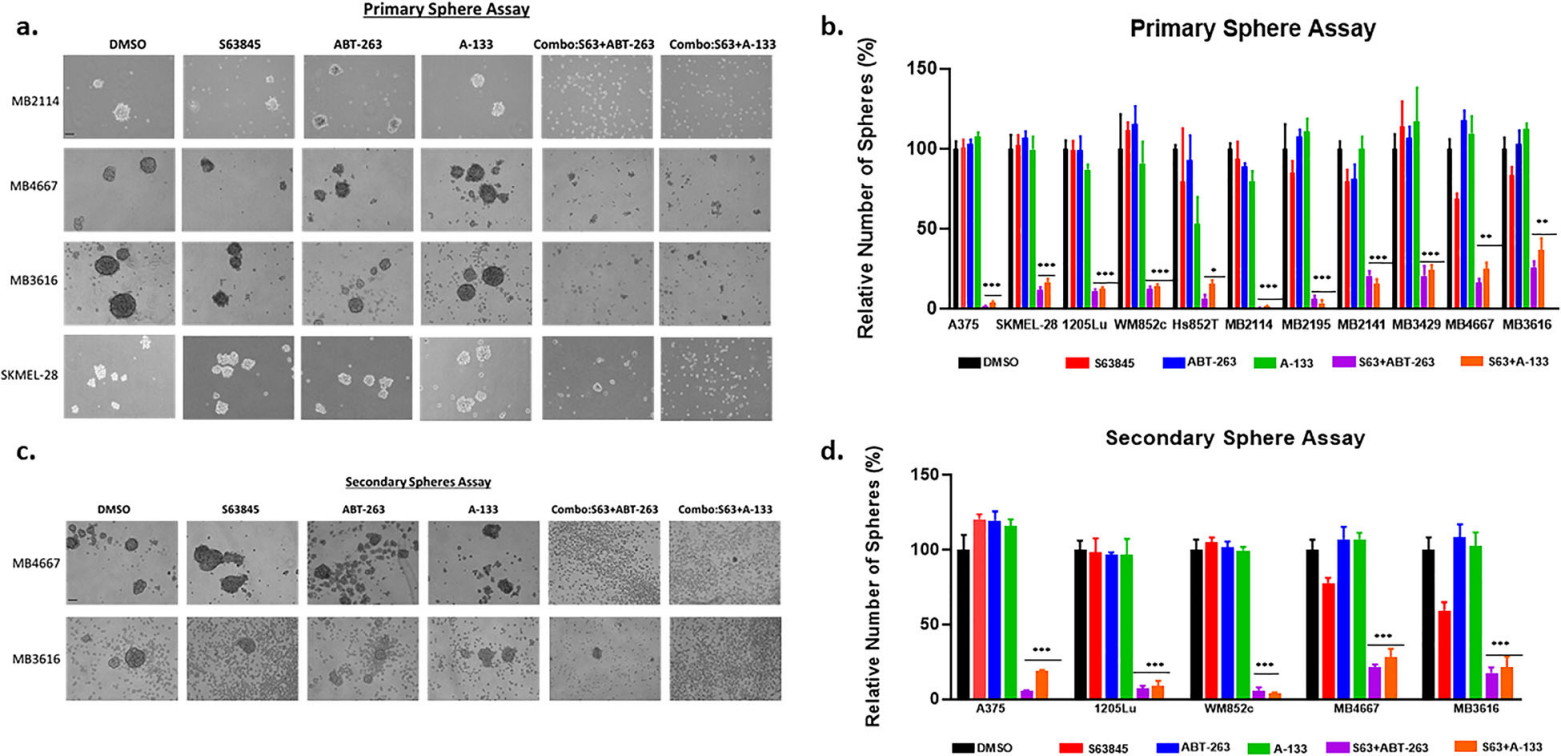
S63845 combined with ABT-263 or A-1331852 killed the resistant heterogeneous MIC population and inhibited the self-renewability. **a**, **b** Melanoma cells were subjected to the primary sphere assay. Spheres were treated with indicated compounds either alone, or in combination, for 48 h, and were then analyzed by bright field microscopy (**a**) and the number of primary spheres quantified (**b**). **c**, **d** The combination treatment also inhibited the formation of secondary spheres (**c**) and quantified data for the number of secondary spheres is expressed as bar graph in (**d**). In all melanoma lines, the combination treatment significantly inhibited sphere maintainence/formation compared with all other treatments (DMSO or single drug). For visual clarity, we marked only the combinational treatments that were significantly different from comparisons with the DMSO and the single-drug treatments. Within each significant combination treatment, we only showed the most significant *p*-value of the comparisons. *Indicates *p* < 0.05; **indicates *p* < 0.01; ***indicates *p* < 0.001. Scale bar = 100 μm. Error bars represent ± SEM.

We aimed to evaluate the therapeutic efficacy of BH3 mimetic combination in the resistant heterogeneous MIC population using surface-marker independent sphere formation assays, as used in many publications^19–26^. The primary sphere assay measures the potency of killing MICs^19,20,27,28^, whereas the secondary sphere assay measures the self-renewal capacity of the MICs after initial treatment^20,27^. In primary sphere culture, bright-field images showed complete disruption of primary spheres after 48 h of drug treatment (Fig. 4a). The combination treatment with S63845+ABT-263 or S63845+A-1331852 significantly reduced the number of primary spheres in several melanoma cell lines (*p* < 0.05) (Fig. 4b), compared with DMSO or single drug. In the secondary sphere assay, combination treatment eliminated almost all sphere formation (Fig. 4c, d) compared to DMSO or single drug treatment (*p* < 0.001) in all cell lines tested. These results suggest that BH3 mimetic combinations may be important in preventing relapse caused by heterogenous MICs.

### Inhibition of both MCL1 and BCLXL was effective in killing diverse types of melanoma cells resistant to current therapies

**Fig. 5:**
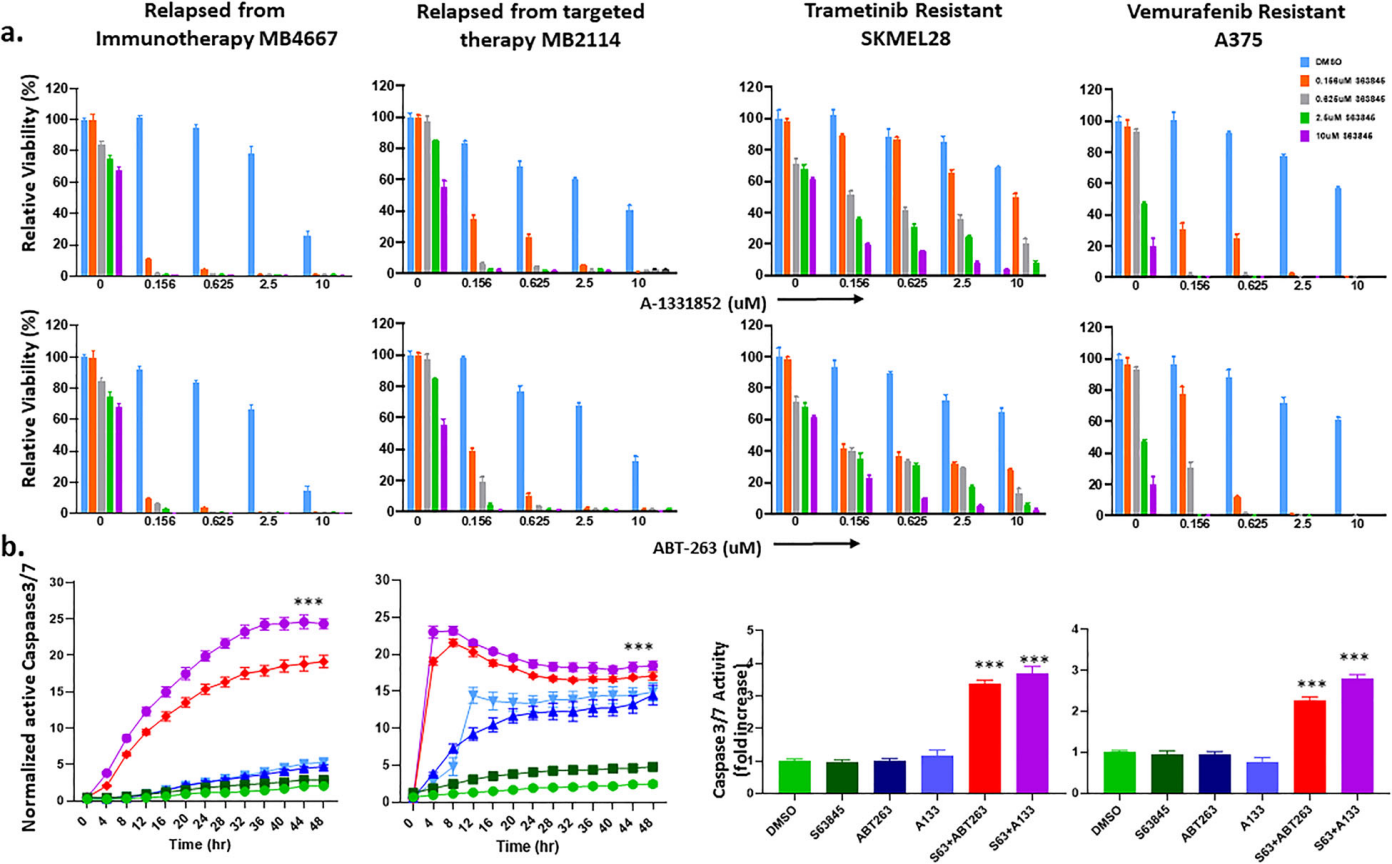
S63845 combined with ABT-263 or A-1331852 were potent to kill melanoma cells resistant to current therapies. Cell lines include patient samples relapsed from targeted therapy or anti-CTLA-4/PD-1 immunotherapy, or those with acquired resistance to a BRAF inhibitor (Vemurafenib) or a MEK inhibitor (Trametinib), which were created using A375 and SKMEL-28 melanoma lines. **a** ATP assay. **b** IncuCyte live cell imaging with active Caspase 3/7 for quantification of apoptosis. Images were acquired using the phase and green fluorescent channels every 4 h for a total of 48 h. For visual clarity, we marked only the combinational treatments that were significantly different from comparisons with the DMSO and the single-drug treatments. Both the combinations (S63845 plus A-1331852 in (**a**), or S63845 plus ABT-263 in (**b**)), significantly (***indicates *p* < 0.001) reduced cell viability and increased apoptosis compared with DMSO or with single drug treated conditions in all melanoma cell lines at sub-micromolar doses. For visual clarity * is not shown in the figure for (**a**). Error bars represent ± SEM. For (b) the same color represent the same treatment condition for the line and the bar graphs.

Currently, immunotherapy is the standard of care for melanoma patients. Although successful for 40% of patients, relapse does occur. BRAF or MEK inhibitors are the first line of treatment for patients with the common *BRAF*^*V600E*^ mutation, however, most show resistance and/or relapse after the initial response. We examined patient-derived cell lines from those who had relapsed from anti-CTLA-4/PD-1 immunotherapy or targeted therapy (MB4667, MB2114 in Fig. 5a and MB3961 in supplementary Fig. 6). Our BH3 mimetic combination therapy (S63845+ABT-263, or S63845+A-1331852) significantly reduced cell viability (*p* < 0.001) in these cells.

To mimic the clinical scenario of patient relapse from targeted therapy, we also used BRAF- and MEK-inhibitor treated melanoma cell lines, resistant up to doses of 5 μM and 200 μM, respectively (Supplementary Fig. 7a). Notably, we found that both MCL1/BCLXL combination treatment (S63845+ABT-263, or S63845+A-1331852) significantly reduced cell viability at sub-micromolar doses (*p* < 0.001) (Fig. 5a and Supplementary Fig. 7b).

To further quantify the effects on apoptosis and proliferation, we conducted IncuCyte live cell imaging of both active Caspase 3/7 and confluency upon drug treatments (Fig. 5b, Supplementary Fig. 8 and 9 and Supplementary Video 4, 5 and 6). In all of the lines relapsed from current therapies, both combination treatments (S63 + A-133 and S63 + ABT-263) significantly increased Caspase 3/7 activation (*p* < 0.001) and decreased proliferation (*p* < 0.001), compared with vehicle or single-drug treatments. These data demonstrated significant induction of apoptosis by both combinations in multiple melanoma cells. These data suggest that combining MCL1 and BCLXL inhibition may be clinically relevant, and should be further explored as a treatment approach for melanomas resistant or relapsed to standard of care treatment.

### NOXA and BIM do not act as significant contributors in combination treatment-induced cell death

**Fig. 6:**
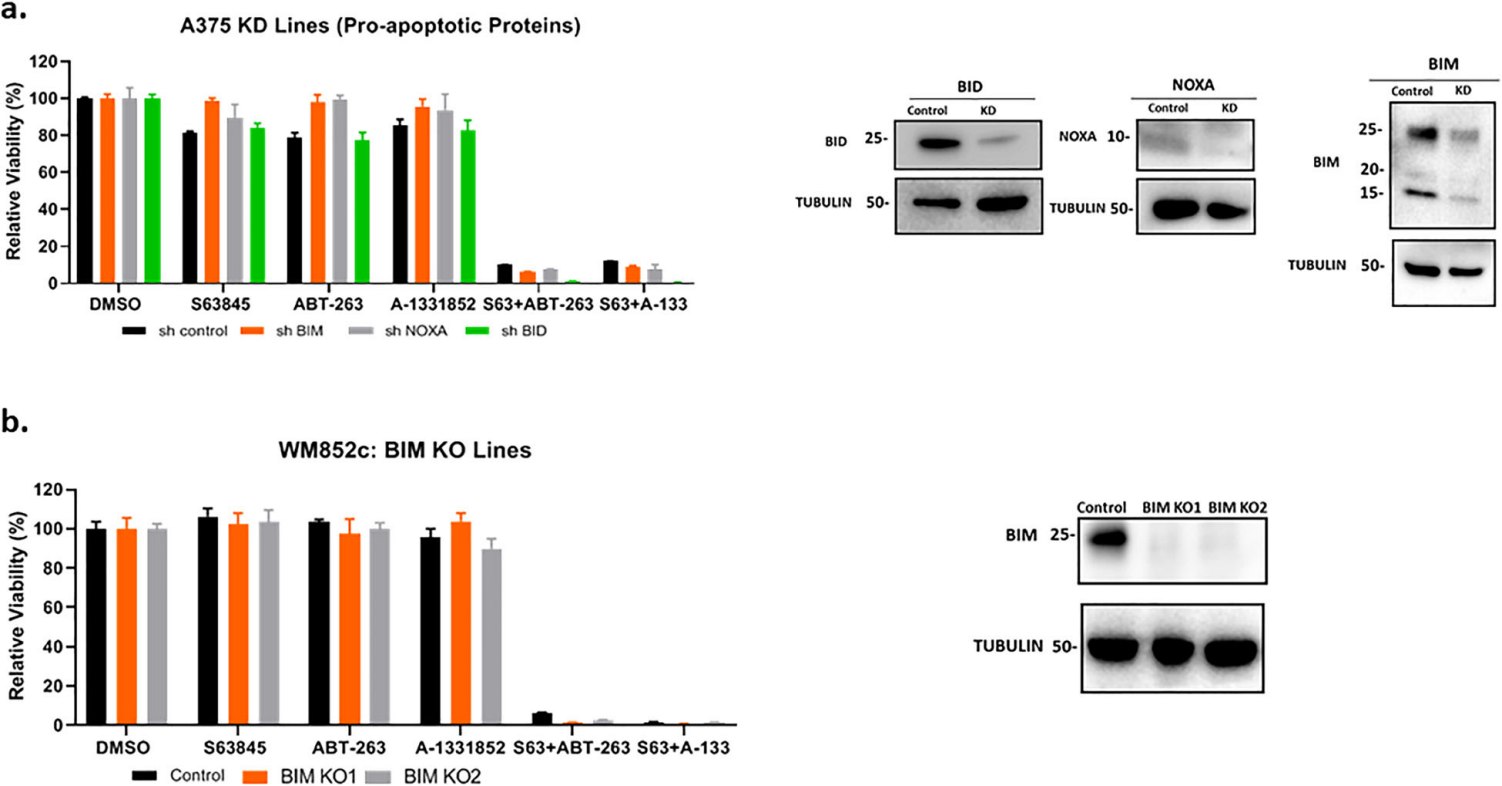
The combination-induced cell death was not dependent on NOXA or BIM. **a**, **b** ATP assay with shRNA mediated KD lines for NOXA, BIM, and BID (**a**) and BIM knockout (KO) lines (**b**) to test if the KD/KO protects against combination-induced cell death. Immunoblot to show the knockdown or knockout effects of NOXA or BIM. Molecular weight markers are in kDa. Error bars represent ± SEM.

Presence of the BH3 only BCL2 family members NOXA and BIM have been shown to be crucial for the killing effects of certain BH3 mimetic treatments^6,11–13,29–32^. We determined whether knockdown or knockout of NOXA and BIM, with shRNAs or CRISPR/Cas9, is necessary for the efficacy of the MCL1/BCLXL combinations (Fig. 6a, b). We did not find significant cell death by silencing these genes (Fig. 6a, b). These results indicate that the mechanisms involved are independent of expression of the pro-apoptotic proteins NOXA and BIM to induce cell death.

We have examined the effects of drug treatments on the main BCL2 anti-apoptotic members by immunoblot (Supplementary Fig. 10). The most consistent alterations upon single-drug treatments of ABT-263 or A-1331852 was that ABT-263 alone slightly increased MCL1. S63845 alone increased MCL1 significantly as reported previously by others^33^. Therefore, our data did not show strong negative feedback loops between BCL2 family members.

### Combinations reduce tumor growth in an in vivo mouse xenograft model

**Fig. 7:**
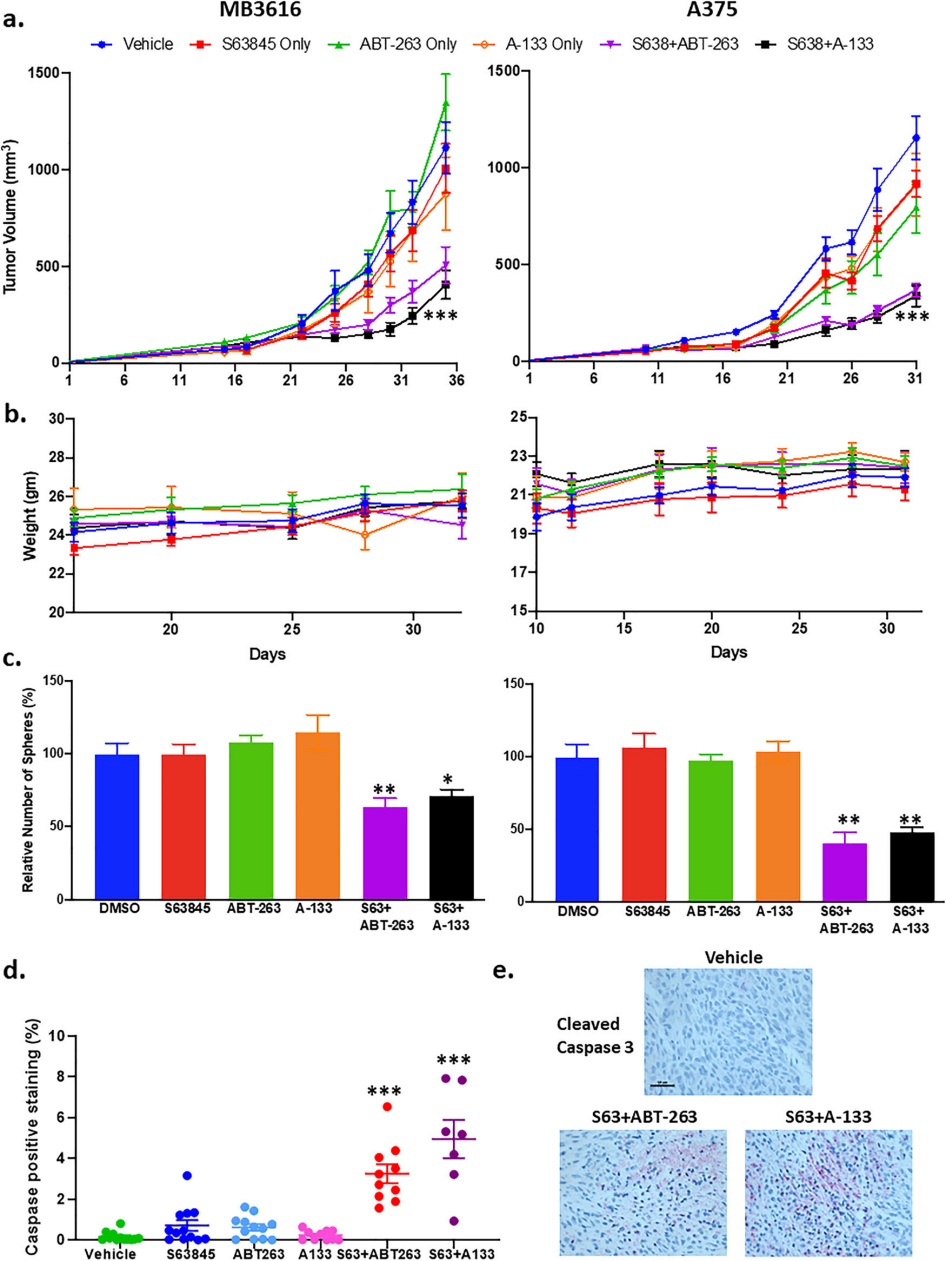
The combination reduced tumor growth in a mouse xenograft model. **a** Tumor volume in mouse xenograft models with patient sample MB3616 and melanoma line A375. Both the combination treatments significantly inhibited the tumor growth compared to vehicle or the single drugs for multiple days. For visual clarify, we marked only the last day. **b** Weight of the mice during the treatment period of the experiment from (**a**). **c** Sphere assays with tumor cells collected at the end of the experiment from (**a**). **d** Quantification of the number of Cleaved Caspase-3-positive area in vehicle, single drug and combination treated mouse tumors. The combination significantly reduced the number of spheres and increased the percentage of Cleaved Caspase-3 positive area compared to vehicle or individual treatments. **e** Representative IHC images of Cleaved Caspase-3 staining from tumor sections derived from mouse xenografts experiments above. Scale bar, 50 μm. *Indicates *p* < 0.05; **indicates *p* < 0.01; ***indicates *p* < 0.001. Error bars represent ± SEM.

We tested the efficacy of MCL1 plus BCLXL combination treatment in a mouse xenograft model. We used human melanoma cell line A375 (*BRAF*^*V600E*^ mutated) and the patient line MB3616 (*NRAS*^*Q61K*^ mutated). Combinations of S63845 with ABT-263/A-1331852 significantly inhibited tumor growth of both lines, compared with control or single drug (*p* < 0.001) (Fig. 7a). We did not see any significant weight loss in the single or combination treated mice at the administered doses (Fig. 7b). Further, the residual tumors from the combination treatment had reduced ability to form secondary spheres compared to single-drug treatment (*p* < 0.05) (Fig. 7c). Immunohistochemistry for Cleaved Caspase-3 (an apoptosis marker) and Ki67 (a proliferation marker) on the tumor sections showed that the combination treatments significantly increased the Cleaved Caspase-3 positive cells (*p* < 0.001) (Fig. 7d, e) and decreased Ki67 positive cells (*p* < 0.01) (Supplementary Fig. 11). These results support that the dual targeting of MCL1 and BCLXL is a promising approach for the treatment of melanoma.

### S64315, the clinical-trial version of S63845, has synergistic effect when combined with BCLXL inhibitors

**Fig. 8:**
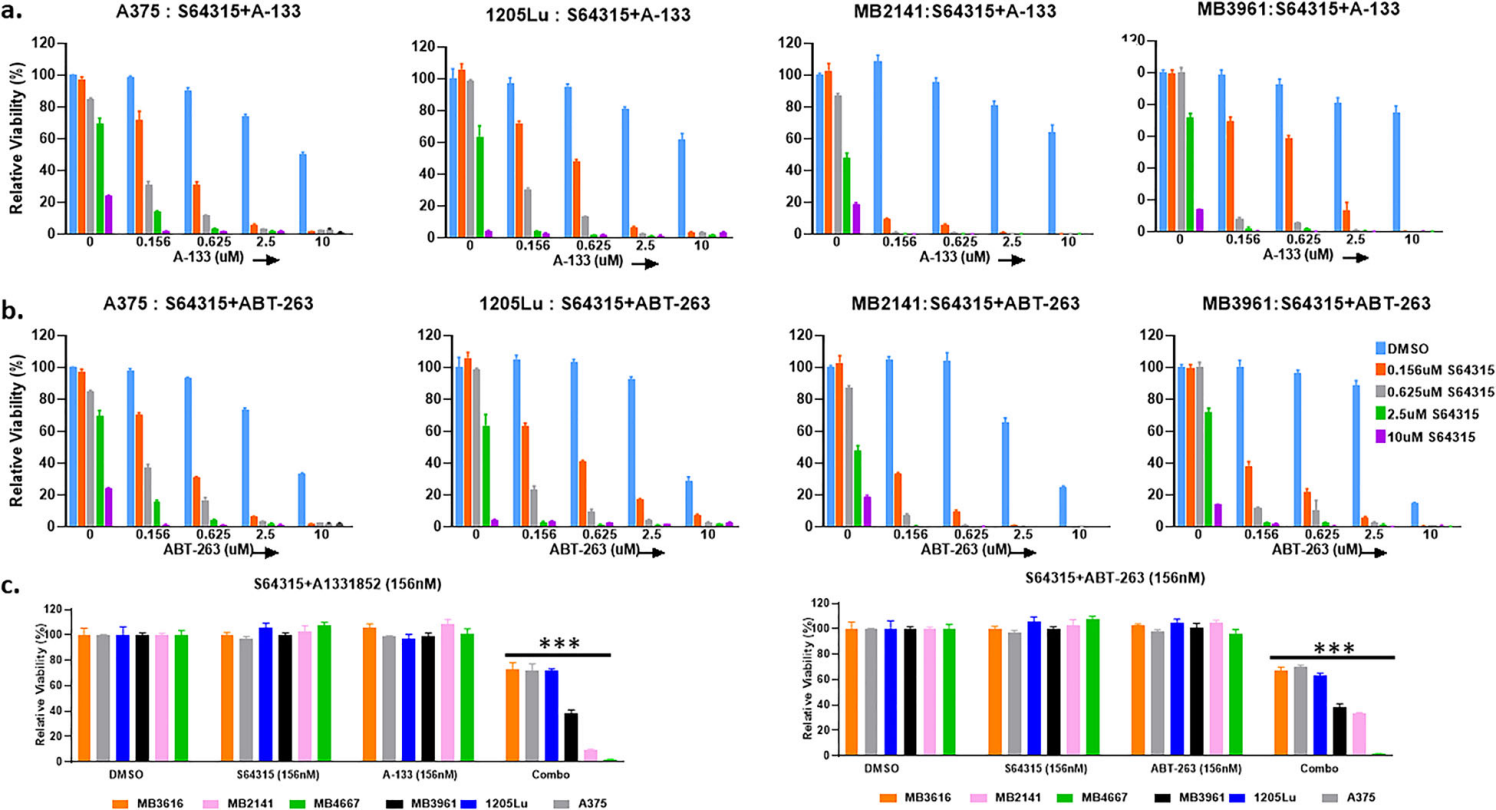
Combination therapy of S64315 (clinical trial version of S63845) with ABT-263/A-1331852 has synergistic effect in treating melanoma samples of diverse genetic backgrounds. **a**, **b** ATP assays of melanoma cell lines and patient samples upon indicated treatments for 48 h. The viability of the DMSO control for each cell line was set to 100%. Both the combinations (S64315+A-1331852 in (**a**); S64315+ABT-263 in (**b**)) significantly (*p* ≤ 0.01) reduced cell viability compared with DMSO or with single drug treated conditions in all melanoma cell lines at sub-micromolar doses. For visual clarity, the * is not shown in the figure. Both the combinations were highly synergistic at sub-micromolar doses. **c** Summary of ATP assay data of six melanoma cell lines and patient samples treated with S64315+A-1331852 or S64315+ABT-263. For **c** all drugs were used at a dose of 156 nM. For visual clarity, we marked only the combinational treatments that were significantly different from comparisons with the DMSO and the single-drug treatments. Within each significant combination treatment, we only show the least significant *p*-value of the comparisons. ***Indicates *p* < 0.001. Error bars represent ± SEM.

S63845 is the parent compound for S64315(MIK665), which is tested in clinical trials for hematopoietic cancers and was recently made commercially available. Thus, we evaluated the efficacy of S64315 in combination with ABT-263/A-1331852 in representative melanoma cell lines and patient samples. Overall, S64315 exhibited similar or slightly better effects than S63845, either alone or in combinations (Fig. 8).

## Discussion

Despite recent advances in treating melanoma, options are still limited for patients without mutations suitable for targeted therapies, for patients who do not respond to immunotherapies, or patients that become refractory to treatment. Developing new treatments for malignant melanoma, therefore, remains an important issue. This study explored an entirely new approach and a new class of compounds that target the anti-apoptotic regulators of melanoma, and thus do not require specific mutations or immunologic activation. This pathway has long been recognized as an important biological process in cancer biology. However, until recently it has been difficult to exploit the pathway in the clinical setting. We and others have demonstrated the efficacy of combining MCL1 inhibitors with other BH3 mimetics targeting multiple BCL2 family proteins to kill melanomas in vitro^10,12,15^. We are the first to demonstrate the efficacy of combining S64315 with ABT-263 in killing melanoma of multiple types, cutaneous and rare. Both drugs are currently in clinical trials, and our data support their potential for combination treatment for patients lacking other options. We are the first to report the pre-clinical efficacy of S64315 (MIK665) in any cancer. The in vivo efficacy of the combinations targeting both MCL1 and BCLXL in multiple mouse xenograft studies (both BRAF mutated and BRAF-WT melanoma lines) provides additional data for moving these strategies to clinical trials.

Our studies demonstrated the efficacy of inhibiting both MCL1 and BCLXL in vitro and in vivo, and show that BH3 mimetics are potent at killing several hard to treat melanomas, including patient derived lines of diverse genetic backgrounds, rare subtypes of melanoma, and resistant or relapsed melanomas. Our data suggest the mutational status of BRAF or NRAS does not predict response. These combinations kill melanoma cells, regardless of their mutation status in BRAF or NRAS, likely because MCL1 and BCLXL are downstream of these common mutations^34,35^. Similarly, our combination treatment significantly killed melanoma cell lines derived from patients with rare subtypes, such as mucosal and acral, suggesting that these compounds may work regardless of melanoma origin. These subtypes often do not have common mutations or a high mutational burden, thus are not likely to respond to the current targeted or immunotherapies. This may be due to the general dependence on anti-apoptotic BCL2 family members, however their exact role needs further examination.

Our results also showed that the combinations killed melanomas derived from patients who had relapsed from current treatments, or melanomas that acquired resistance to targeted therapies during in vitro selections. Strikingly, combining the direct and potent MCL1 inhibitor S63845 with A-1331852 or ABT-263, resulted in exceptional killing in relapsed or resistant melanomas at below 200 nM (Fig. 5). The potency is especially impressive, as the older generation of BH3 mimetics required doses greater than 3 μM for similar effects^11–13,15^. Combinations with the newest generation of MCL1 inhibitor had more than 10-fold increase in potency, improving the likelihood of achieving an effective dose in clinical trials. Taken together, these combinations, targeting MCL1 plus BCLXL, offer alternative options for many difficult-to-treat melanomas, and should be tested as a treatment for patients with resistant or relapsed disease.

Our knockdown or knockout experiments also show that combination treatments against MCL1 and BCLXL eliminate the need for the pro-apoptotic proteins NOXA and BIM. This is similar to our previous observation on the effects of combining an early generation of MCL1 inhibitor (A-1210477) with ABT-263^15^. The knockdown or knockout of BH3-only proteins BIM or NOXA is likely needed for indirect inhibition of MCL1; however, the newest drugs are direct and potent, removing any need for the pro-apoptotic activity from BIM or NOXA. These data are consistent with the Displacement Model of apoptosis, which states that apoptosis is triggered without BH3-only activators of apoptosis (such as BIM or NOXA), if the major pro-survival BCL2 family members, such as MCL1 and BCLXL in melanoma, are inhibited all at once^36^. This hypothesis implies that a cell is primed for apoptosis, but is held in check by pro-survival proteins; inhibiting pro-survival BCL2 proteins sends the cellular machinery to its default death pathway. These data suggest that in tumors, the lack of expression of BH3-only pro-apoptotic proteins would not prevent them from responding to the combinations of S63845 plus ABT-263 or A-1331852, providing further support that these combinations are promising therapies for hard to treat cancers.

Side-effects need to be considered for any treatment that targets the general pro-survival factors MCL1 and BCLXL^37,38^. ABT-263 (pan BCL2) by itself causes dose-dependent thrombocytopenia^39^, and MCL1 inhibition can cause adverse effects on hematopoietic and lymphoid cells^37,38^. Therefore, in combination, MCL1 plus BCLXL inhibitors may be especially toxic for hematopoietic cells^40–43^, especially with high dosages and aggressive schedules^38^. Understanding and managing toxicity will be a key goal prior to moving this promising combination into human trials.

We are the first to demonstrate the in vivo efficacy of targeting both MCL1 and BCLXL simultaneously in multiple mouse xenograft studies (both BRAF mutated and BRAF-WT melanoma lines). We carefully chose an in vivo treatment scheme that minimizes toxicity, a long-standing concern in co-inhibition of MCL1 plus BCLX^10,38,44^. Weeden et al. reported acute liver toxicities when S63845 was combined with A-1331852 and suggested that further refinement of the therapeutic window of each drug is needed for successful in vivo treatment^44^. It has been reported that 5 consecutive day dosing with S63845 at 12.5–40 mg/kg, or 21 consecutive days of ABT-263 at 100 mg/kg is well tolerated^33,43,45,46^. Using a MCL1 inhibitor at 50–100 mg/kg and 5–7 days/week resulted in minimal toxicity in mice, when combined with the BCL2 inhibitor ABT-199^42,43^. However, so far, no studies successfully tested the clinically available MCL1 inhibitors in combination with ABT-263 in vivo. Based on our multiple pilot studies and extensive literature search, we administered S63845 at 25 mg/kg for only 2 days per week and ABT-263/A-1331852 at a dose of only 10 mg/kg for two days per week. Decreasing the dose and frequency of treatment appears to be the key in overcoming toxicity and maintaining potency. Necropsy showed no obvious toxic side effects. Moreover, these treatments did not affect mouse body weight dramatically (Fig. 7b). Overall, these data suggest the combination treatments are tolerable. Our dosing was at least 5–10 times less than the reported studies that combine a MCL1 inhibitor with ABT-199, and our approach showed significant tumor shrinkage with no obvious toxicity. Our results provide a starting point for improving dosing and timing prior to human trials. If cancer cells are more dependent on the BCL2 pro-survival factors relative to normal cells^35^, then the potent synergy of the combinations with refined dosing and frequency schedules that kill melanoma cells without significant side-effects is feasible. In future studies, a wide dose range of drugs and more specific drug delivery approaches, such as intratumoral administration or use of nanoparticles for drug delivery, should be evaluated.

Finding predictive biomarkers to identify patients likely to respond to combination therapy with MCL1 and BCLXL inhibition is essential. In an attempt to classify patients as responders or non-responders, we are examining BCL2 family protein expression prior to treatment. Specifically, we correlated the basal expression of BCL2 proteins with response to combination treatment with MCL1 and BCLXL inhibitors. The Pearson correlation analysis indicated that BCLXL expression is the best predictor for response to the combination treatments (data not shown). This work therefore provides a framework for further testing BCLXL as a biomarker.

It is possible that standard of care therapies induce resistance by altering the expression of the BCL2 family members, further making BH3 mimetics an attractive treatment option in relapsed patients. For example, Montero and colleagues recently showed that targeted therapies induce a MCL1 dependency in surviving tumor cells, in melanoma or other solid tumors^47^. The involved mechanism is through induced loss of NOXA, an endogenous inhibitor of MCL1^47^. In addition, MCL1 and BCLXL can be induced as part of the adaptive response in melanoma cells to various triggers, including targeted therapies^48^. Furthermore, the upregulation of MCL1, BCL2, and/or BCLXL is reported after the addition of targeted therapies such as MAPK inhibitors in vitro^47,49–52^. Lastly, BCLXL is upregulated in the tumor microenvironment of both mantle cell lymphoma and follicular lymphoma^53^, and the tumor microenvironment is a crucial factor in determining response to immunotherapy^54^. If this pattern holds true, combination treatments against MCL1 and BCLXL are an especially attractive therapy against advanced melanomas.

In summary, our data strongly indicate that combination treatment targeting both MCL1 and BCLXL may provide a new and novel therapeutic option for patients with advanced melanoma. This combination has the distinct advantage over currently available treatments in that it is not dependent upon specific activating mutations or immunologic activation. These agents are already in clinical trials in other diseases, and we anticipate their rapid introduction into human melanoma studies.

## Materials and methods

### Reagents and drug treatments

S63845, S64315, A-1331852, and ABT-263 were purchased from MedChem Express (Monmouth Junction, NJ) or from Selleck Chem (Houston, TX). All drugs were administered at a dose range of 0.156–10 μM for the cell viability assays. For all other assays, drug treatments of 0.156 µM or 0.625 µM were used, unless otherwise mentioned. Drug treatments were 48 h in duration for all in vitro assays.

### Cell proliferation and apoptosis assays

Cells undergoing drug treatment were monitored for proliferation and apoptosis using the IncuCyte S3 Live-Cell Analysis System (Sartorius/Essen Bioscience). Depending on the cell line, 3000–7000 cells were seeded per well in a 96-well tissue culture plate 24 h prior to drug addition. Cells were maintained at 37 degrees Celsius, 5% CO_2_ in RPMI media with 10% FBS and 1% penicillin–streptomycin. Cells were treated with 0.625 μM single agent S63845, ABT-263, A-1331852, or the specified combination. All treatments were done in triplicate wells. At the time of treatment, IncuCyte Caspase 3/7 Green Apoptosis Assay Reagent (#4440, Sartorius) was added to treatment media per the manufacturer’s instructions. Images were acquired using the phase and green fluorescent channels every 4 h for a total of 48 h. Graphs and images were generated using the IncuCyte Software (v2019B). The confluency was used as a readout for proliferation. Active Caspase 3/7 was normalized by the confluency in each well, which was calculated as the ratio of counts in the green channel versus counts in the bright field channel.

For the experiment with A375 and SKMEL-28 drug resistant lines (Fig. 5), the cells were treated in the same way as described above and the level of Caspase 3/7 was measured by recording fluorescence using a multimode plate reader (Synergy 2 Biotek). The data was plotted and analyzed using GraphPad Prism 6 software.

### Mouse xenograft studies

The Institutional Animal Care and Use Committee (IACUC) of the University of Colorado Denver approved all animal experiments (protocol number 318). NCRNU nude mice, 6–8 weeks of age, were injected subcutaneously in each flank with a 100ul suspension of 2–3.5 million cells in 50% BD Matrigel Matrix, High Concentration, Growth Factor Reduced (BD Biosciences), prepared according to the manufacturer’s protocol. Drug treatments were begun after tumors were palpable. Mice were randomly divided into six treatment groups consisting of at least 8 tumors each group: (1) vehicle only, (2) S63845 only, (3) ABT-263 only, (4) A-1331852 only, (5) S63845 + ABT-263 and (6) S63845 and A-1331852. S63845 and ABT-263/A-1331852 were administered at 25 mg/kg and 10 mg/kg, respectively. ABT-263 and A-1331852 were prepared according to the protocol described previously^45,46,55^. S63845 was prepared by dissolving the drug in 2% kolliphore and 98% sterile PBS. The solution was vortexed and sonicated at room temperature. ABT-263, A-1331852 or vehicle was administered twice weekly for 21 days via oral gavage. S63845 or vehicle was administered via tail-vein or intraperitoneal injection twice weekly for three weeks. To minimize cytotoxicity, drugs were administered on different days. Mice were weighed daily, and tumor volume was measured every 2 days with digital calipers. The following formula was used to calculate tumor volume: tumor volume (mm^3^) = (length × width^2^)/2. At the end of the experiment, the mice were euthanized, and tumors were collected for sphere assays and immunohistochemistry studies.

### Immunohistochemistry (IHC)

The mouse tumors were fixed in 4% paraformaldehyde for 24 h and dehydrated using 70% alcohol at 4 °C. The samples were then paraffin embedded and sectioned by the CU Histology Core. Tissues were sectioned at 4 *μ*m thick sections and dried onto microscope slides and stored at RT until staining. The immunohistochemistry was conducted as described in ref. ^56^. Briefly, staining was done in a Dako Autostainer, and slides were incubated in Dual Endogenous Enzyme Block (#S2003; Dako/Agilent) for 10 min, and in protein free blocking solution (#X0909; Dako/Agilent) for 20 min. Slides were then incubated in primary antibody for 60 min at room temperature (Cleaved Caspase-3, 1:200, #9664 Cell Signaling Technology; and Ki67, 1:100 #RM-9106-S1, Thermo Fisher Scientific). Stains were developed with Vulcan Fast Red (VFR #FR805S BioCare Medical) for 15 min. Slides were washed using 1× Wash Buffer after incubation with each reagent and with dH_2_O following incubation with VFR. Slides were counterstained with Hematoxylin (#S3301 Dako) for 10 min. The quantification procedure was adapted from Loewe et al., paper^57^. All image capture and image quantification were done by individuals blind to the treatment conditions. For Ki67 stained slides, two to three representative 40x magnified images per tumor section were taken from a central area of uniform staining, using an upright microscope (Leica DM 2500) with camera (Leica DFC 500). The Ki67 positive cells were counted by two individuals. For Cleaved Caspase-3 stained slides, two high power fields were randomly photographed from the area of the tissue section with Caspase-3 staining. The percentage of Caspase-3 positive area out of the total area was evaluated using NIS-Elements BR -software (Ver4.13) by two individuals blinded to the treatment conditions.

### Statistical analysis

All graphs for the ATP and sphere-forming assays, as well as statistical analyses were created in GraphPad Prism 6 software. Statistically significant differences among experimental conditions were evaluated by *t*-test or one-way ANOVA followed by Tukey post-hoc tests to identify significantly different comparisons among the groups. For mouse xenograft studies, two-way ANOVA (mixed model) of treatment groups and days, followed by Tukey post-hoc tests was used to identify significance. Error bars represent mean value with standard error of mean. Sample size and replicates are indicated in each method described above. All graphs indicating multiple repeated measurements are presented as mean values with standard error of mean.

## Supplementary information

Supplementary Figure 1.

Supplementary Figure 2.

Supplementary Figure 3.

Supplementary Figure 4.

Supplementary Figure 5.

Supplementary Figure 6.

Supplementary Figure 7.

Supplementary Figure 8.

Supplementary Figure 9.

Supplementary Figure 10.

Supplementary Figure 11.

Supplemental Table-1

Supplemental Table-2

Supplementary Figure legends-clean copy

Supplementary materials and methods (clean version)

Supplementary Movie 1-SKMEL28-DMSO

Supplementary Movie 2-SKMEL28-S63+ABT-263

Supplementary Movie 3-S63+A-133

Supplementary Movie 4-MB4667-DMSO

Supplementary Movie 5-MB4667-S63+A133

Supplementary Movie 6-MB4667-S63+ABT-263
