# Supplementary material for: MCL1 inhibitors S63845/MIK665 plus Navitoclax synergistically kill difficult-to-treat melanoma cells
Source: Cell Death Dis. 2020 Jun 8;11(6):443. doi: 10.1038/s41419-020-2646-2 (PMC7280535; doi:10.1038/s41419-020-2646-2)
Supplement: Supplementary file 1 — Supplementary Figure 1. [file 41419_2020_2646_MOESM1_ESM.docx]

**Supplementary Table 2: IC50 value of the Patient sample lines and cell lines**

| **Patient Line** | **S63845 (μM)** | **A133 (μM)** | **ABT-263 (μM)** |
| --- | --- | --- | --- |
| MB4667 | 14.19 | 7.197 | 5.05 |
| MB2114 | 10.71 | 7.467 | 7.077 |
| MB3429 | 8.854 | 11.68 | 7.12 |
| MB3616 | 8.107 | 12.43 | 10.09 |
| MB1692 | 7.415 | 14.75 | 8.581 |
| MB3447 | 5.49 | 4.708 | 5.662 |
| MB3961 | 2.907 | 14.18 | 6.689 |
| MB2141 | 2.67 | 12.61 | 6.533 |
| MB2724 | 3.242 | 2.152 | 1.692 |
| MB2195 | 2.37 | 10.8 | 8.469 |

| **Cell Lines** | **S63845 (μM)** | **A133 (μM)** | **ABT-263(μM)** |
| --- | --- | --- | --- |
| A375 | 6.335 | 19.35 | 7.318 |
| 1205Lu | 7.195 | 13.87 | 7.415 |
| 451Lu | 7.593 | 8.387 | 6.776 |
| WM852c | 7.811 | 11.68 | 4.634 |
| SKMEL-28 | 4 | 17.79 | 14.76 |
| Hs294T | 2.901 | 11.43 | 6.835 |
