# Supplementary figures and images for: MCL1 inhibitors S63845/MIK665 plus Navitoclax synergistically kill difficult-to-treat melanoma cells
Source: Cell Death Dis. 2020 Jun 8;11(6):443. doi: 10.1038/s41419-020-2646-2 (PMC7280535; doi:10.1038/s41419-020-2646-2)

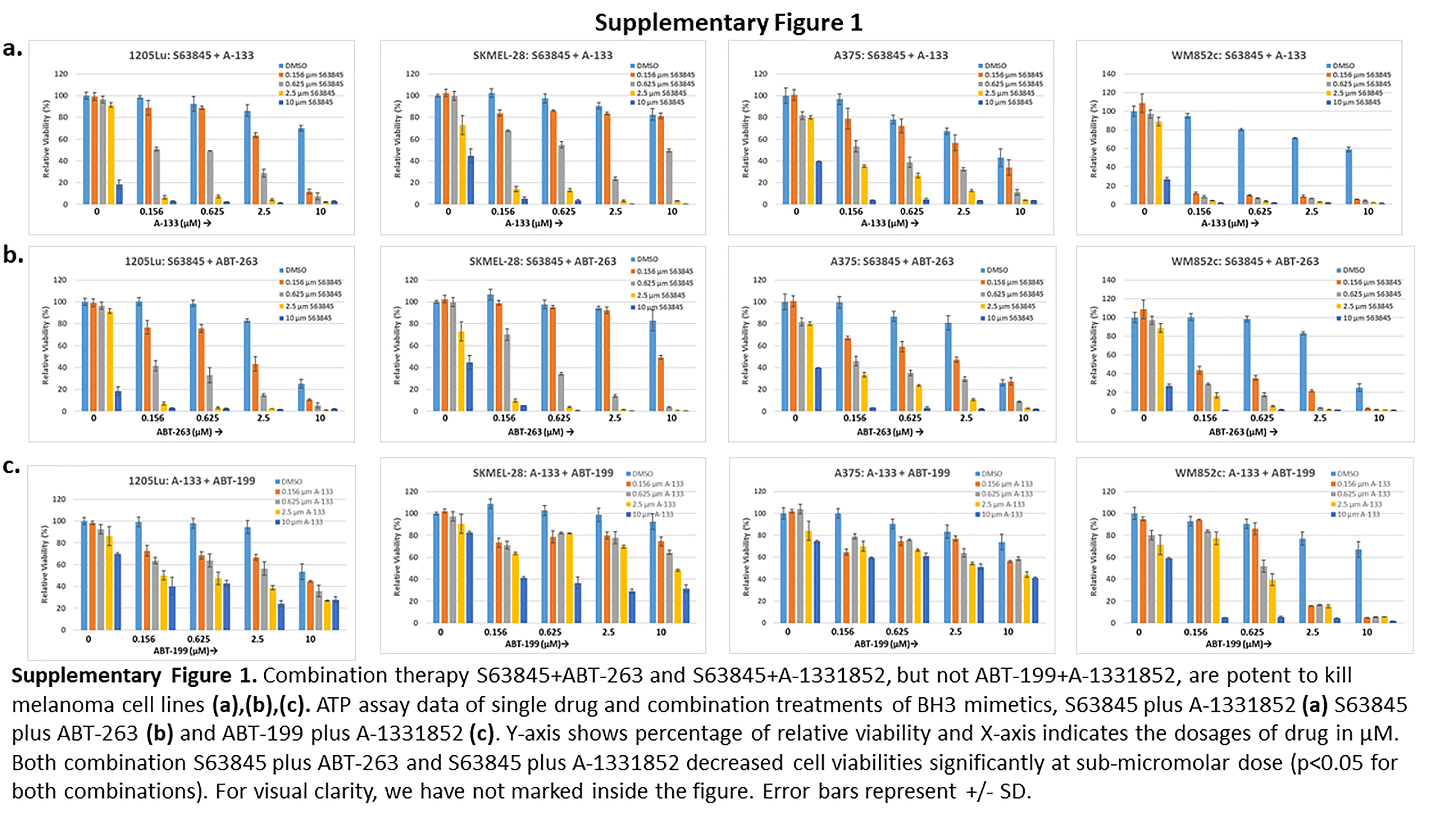

Supplement: Supplementary file 2 — Supplementary Figure 2. [file 41419_2020_2646_MOESM2_ESM.tif]

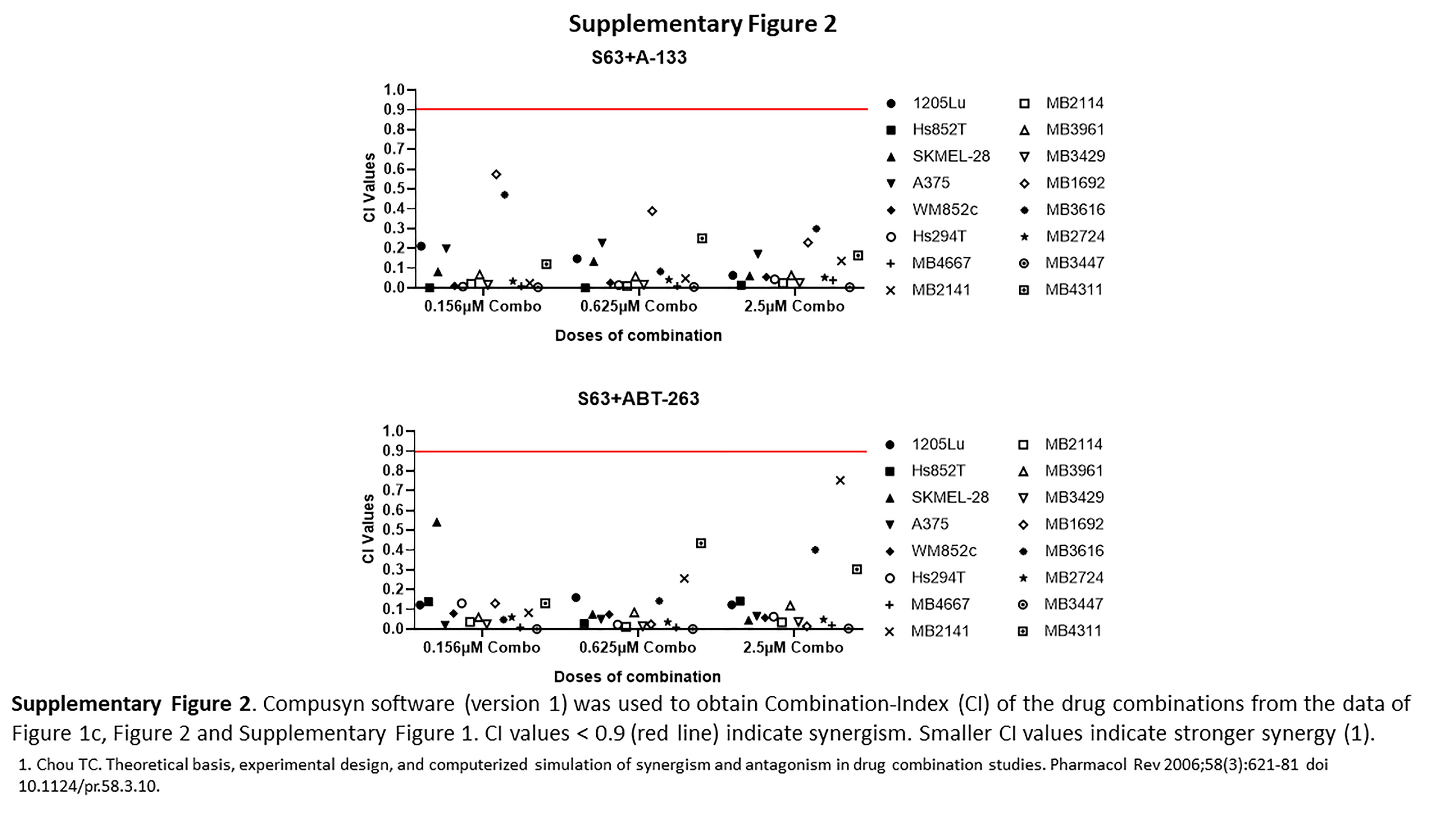

Supplement: Supplementary file 3 — Supplementary Figure 3. [file 41419_2020_2646_MOESM3_ESM.tif]

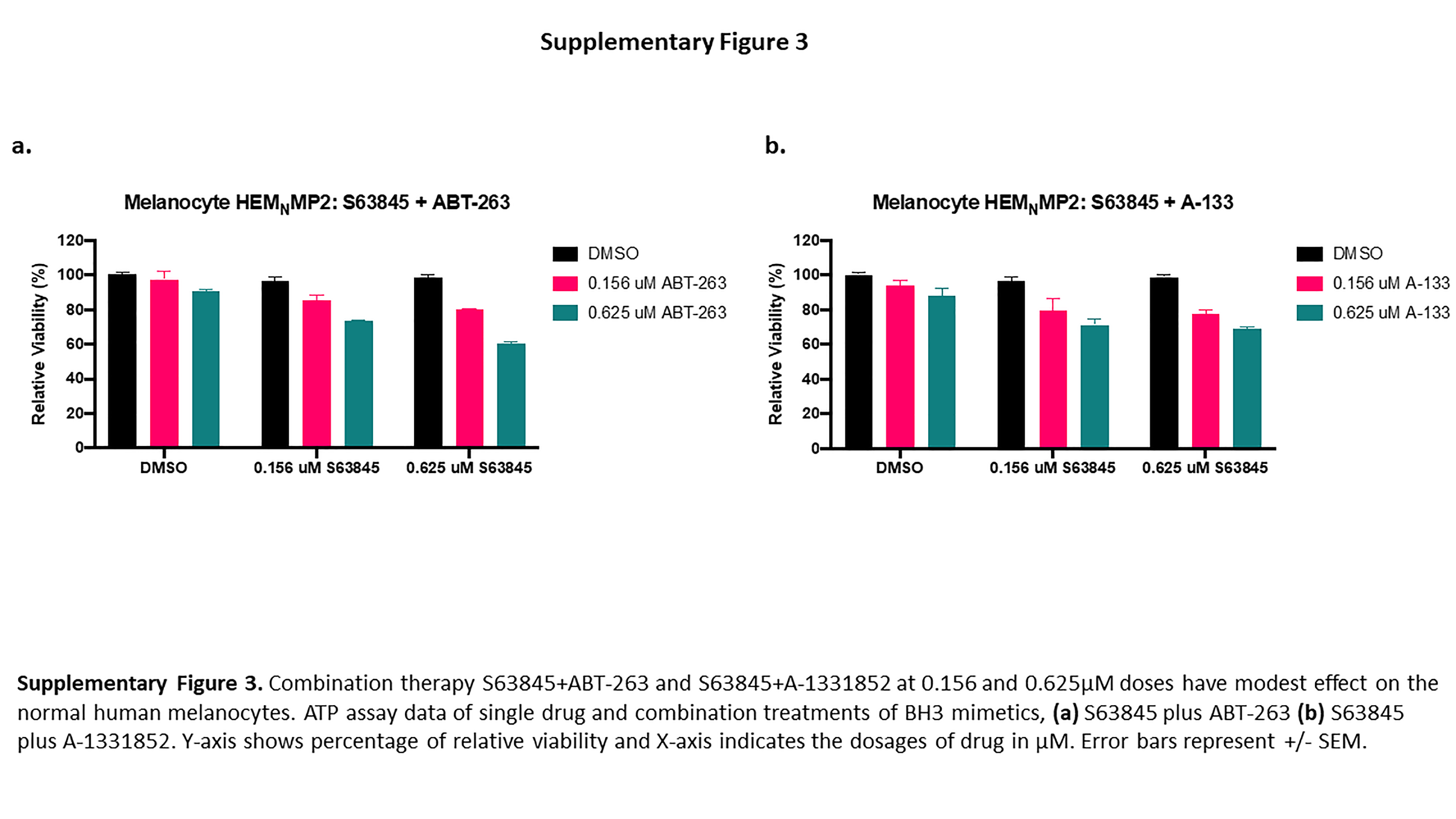

Supplement: Supplementary file 4 — Supplementary Figure 4. [file 41419_2020_2646_MOESM4_ESM.tif]

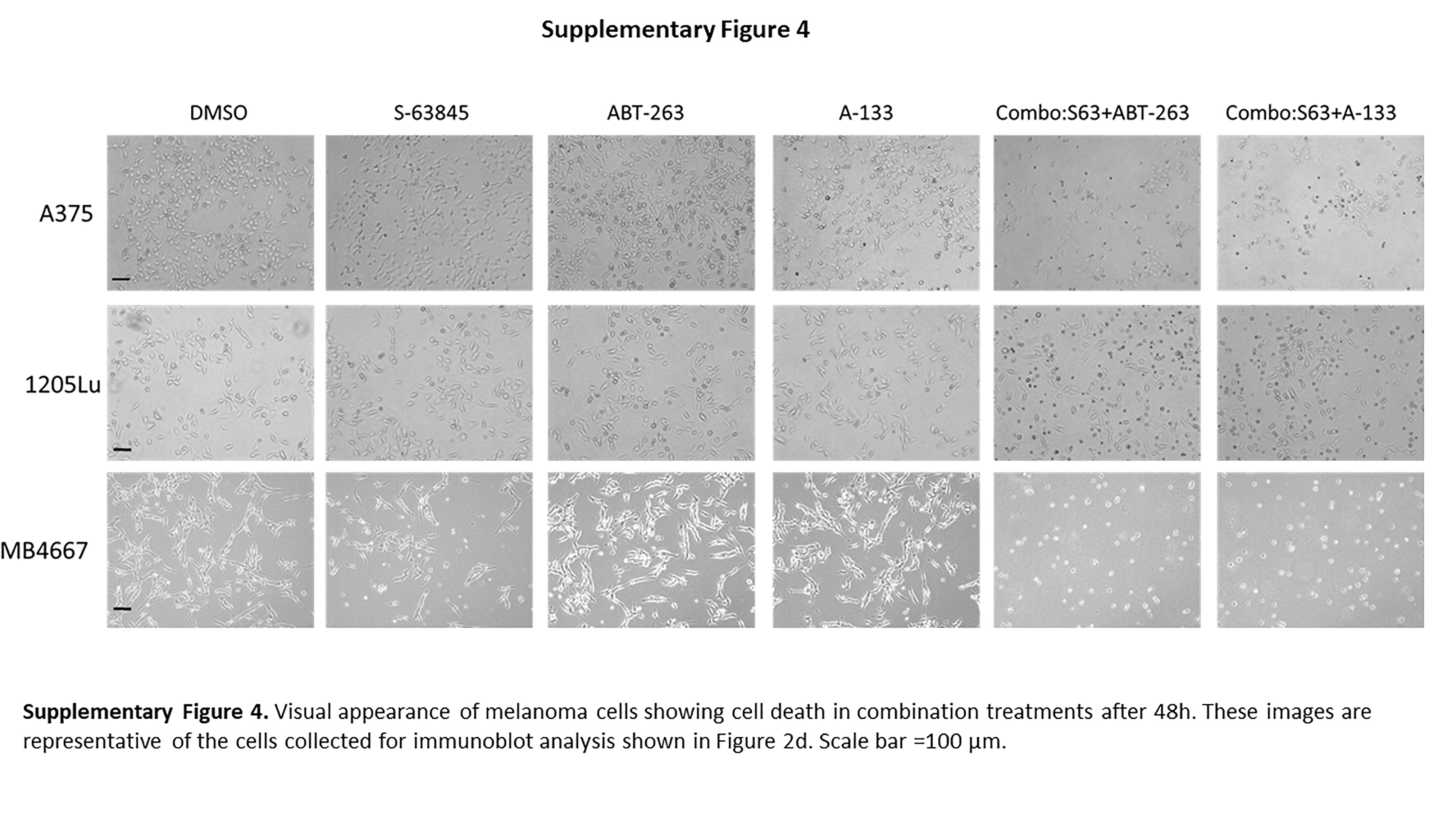

Supplement: Supplementary file 5 — Supplementary Figure 5. [file 41419_2020_2646_MOESM5_ESM.tif]

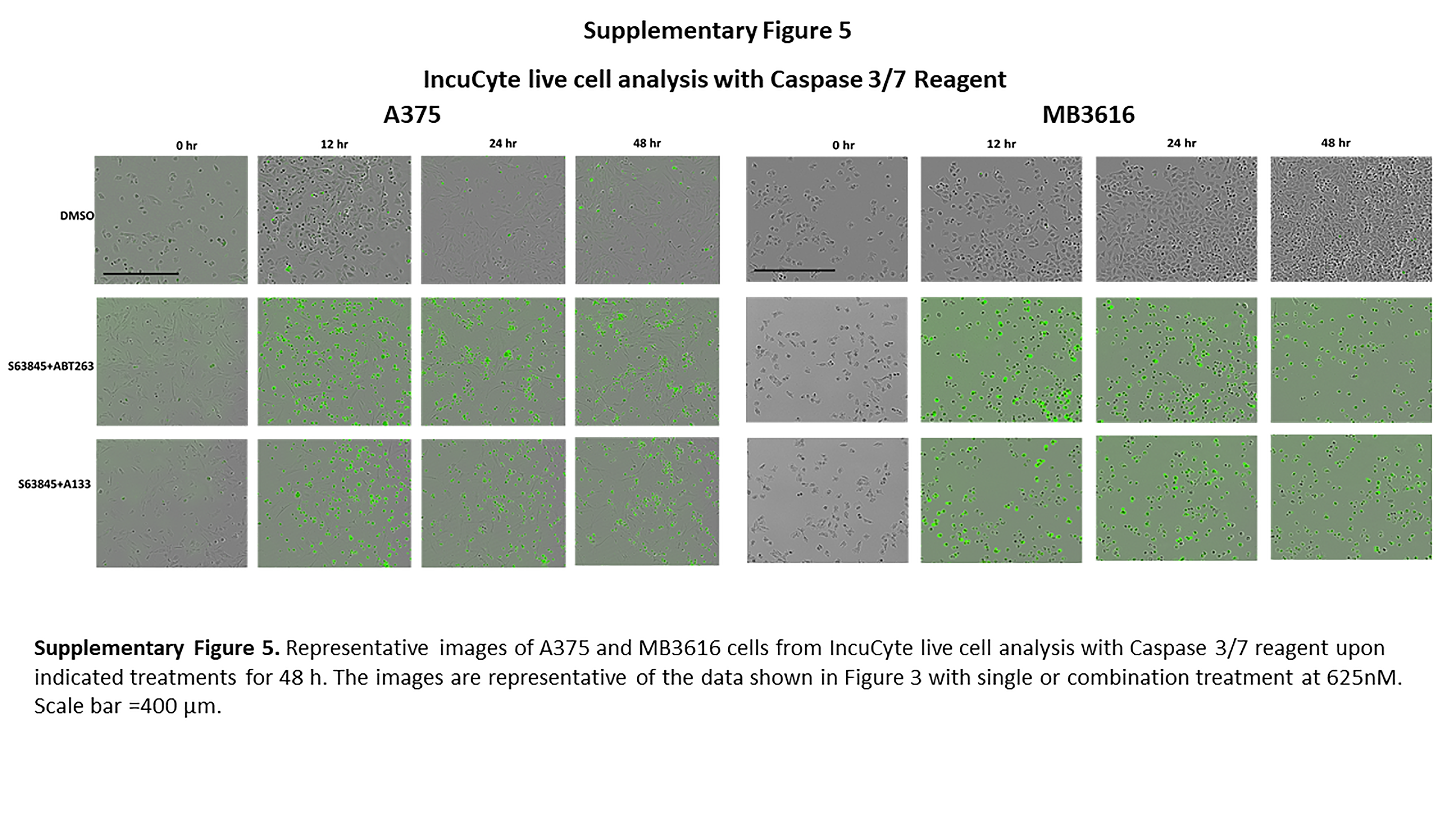

Supplement: Supplementary file 6 — Supplementary Figure 6. [file 41419_2020_2646_MOESM6_ESM.tif]

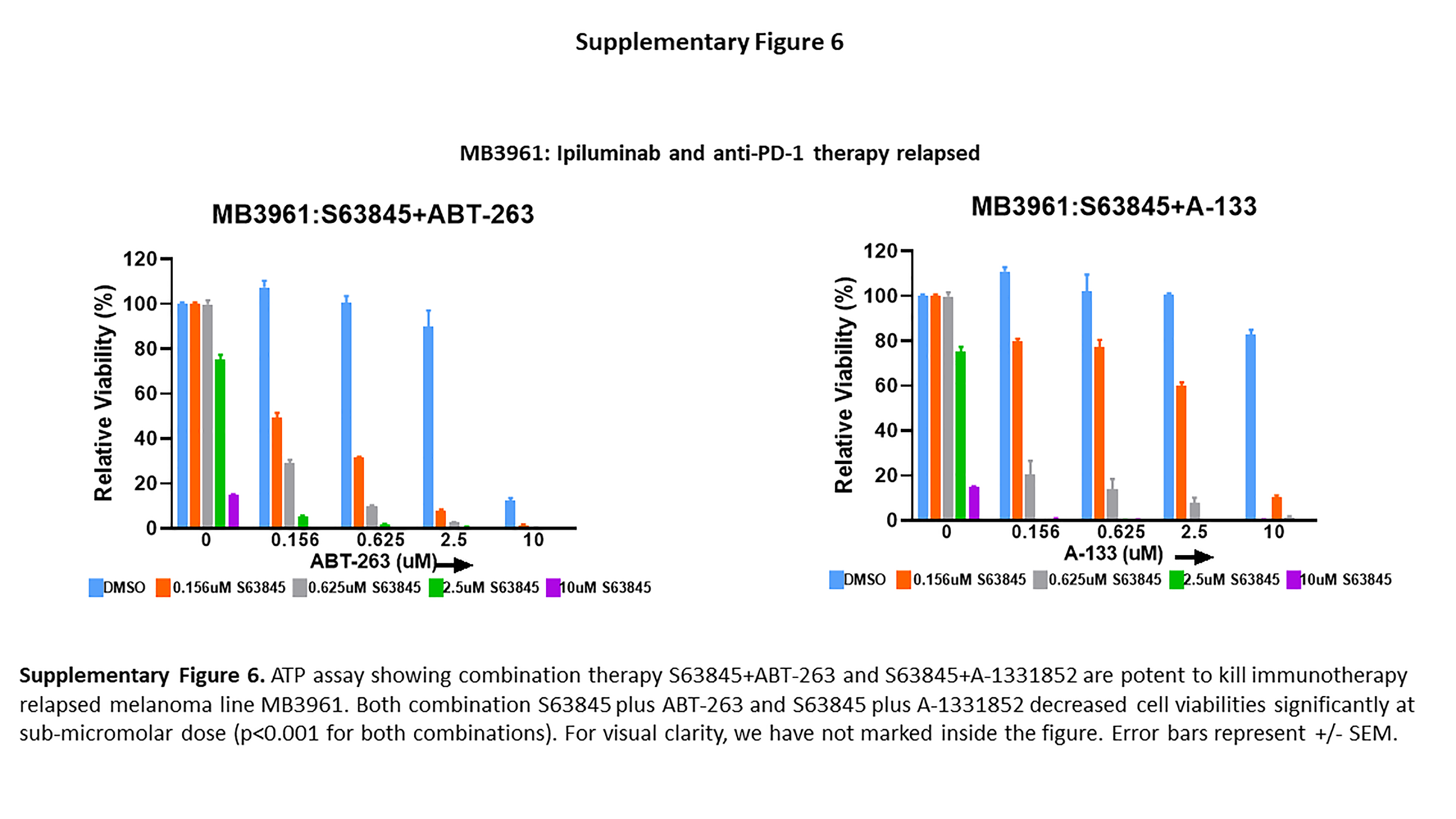

Supplement: Supplementary file 7 — Supplementary Figure 7. [file 41419_2020_2646_MOESM7_ESM.tif]

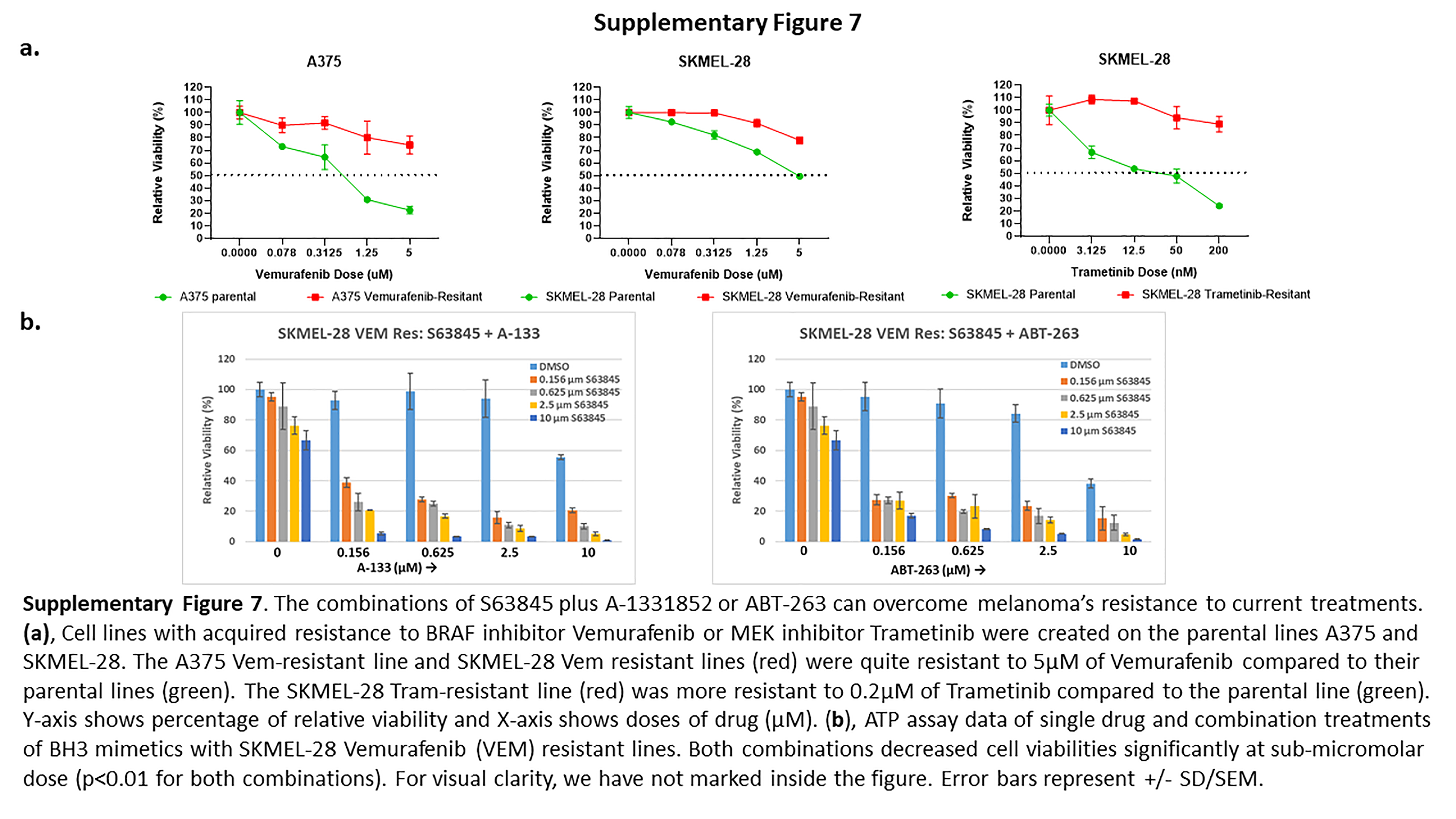

Supplement: Supplementary file 8 — Supplementary Figure 8. [file 41419_2020_2646_MOESM8_ESM.tif]

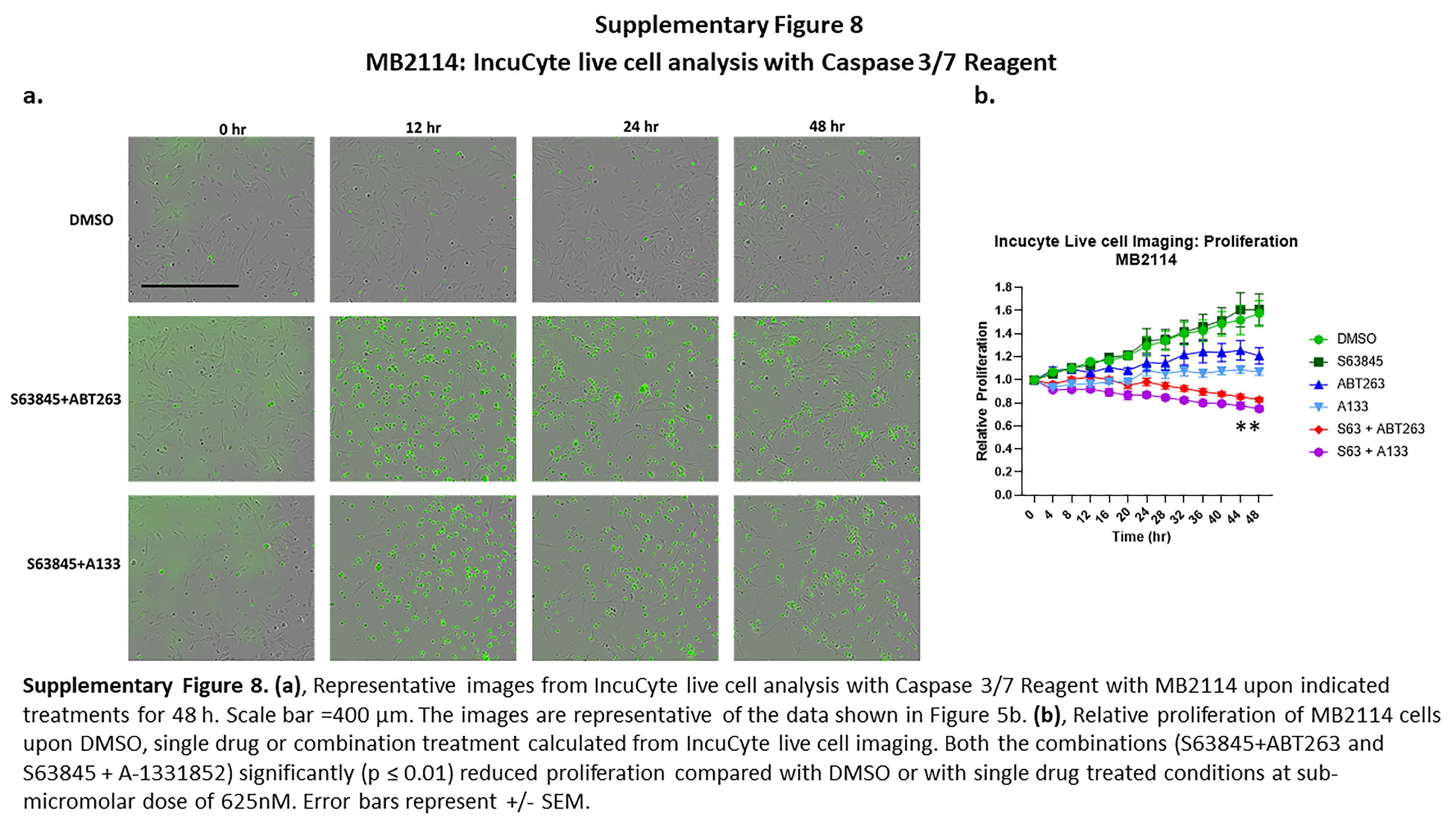

Supplement: Supplementary file 9 — Supplementary Figure 9. [file 41419_2020_2646_MOESM9_ESM.tif]

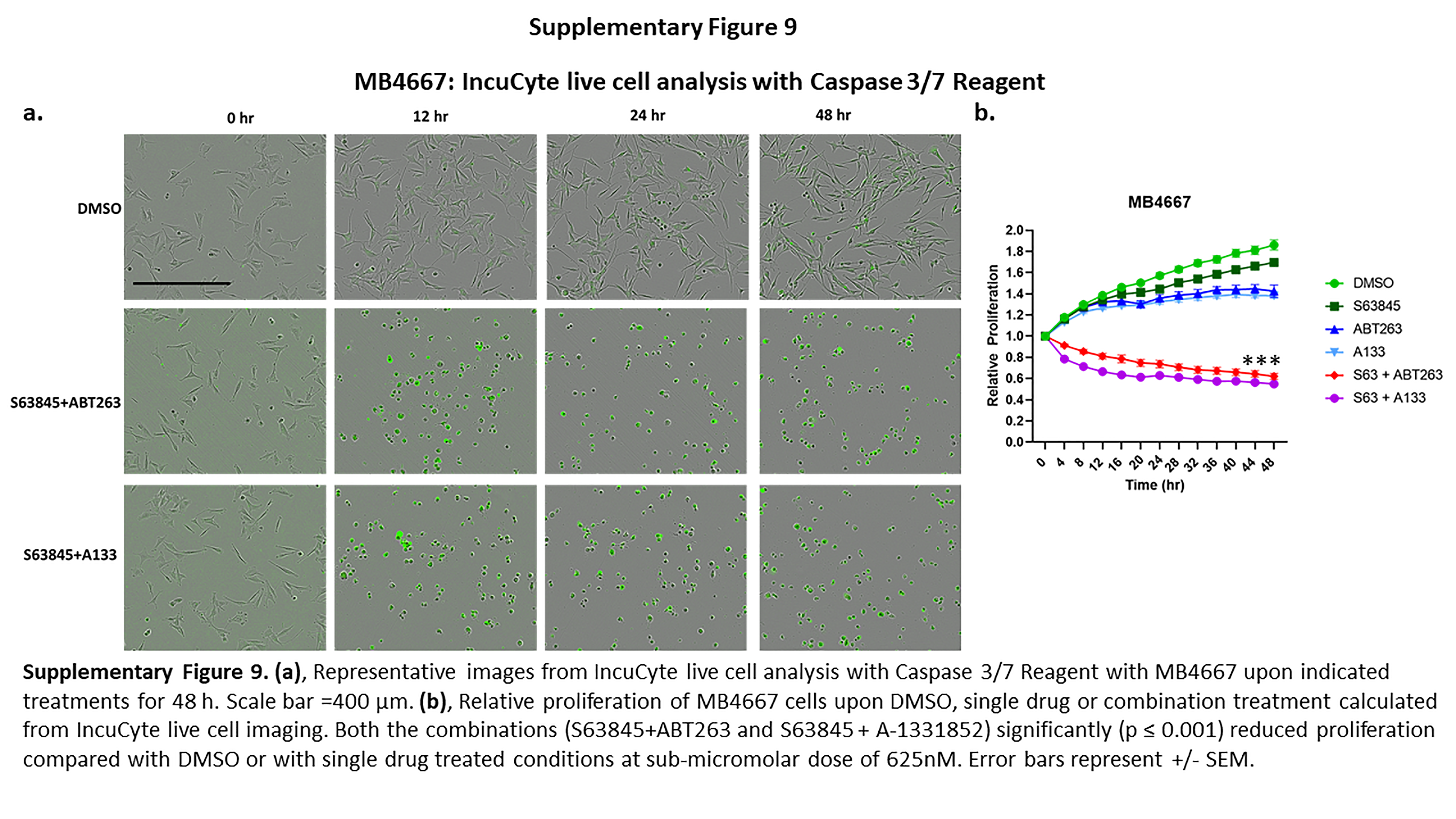

Supplement: Supplementary file 10 — Supplementary Figure 10. [file 41419_2020_2646_MOESM10_ESM.tif]

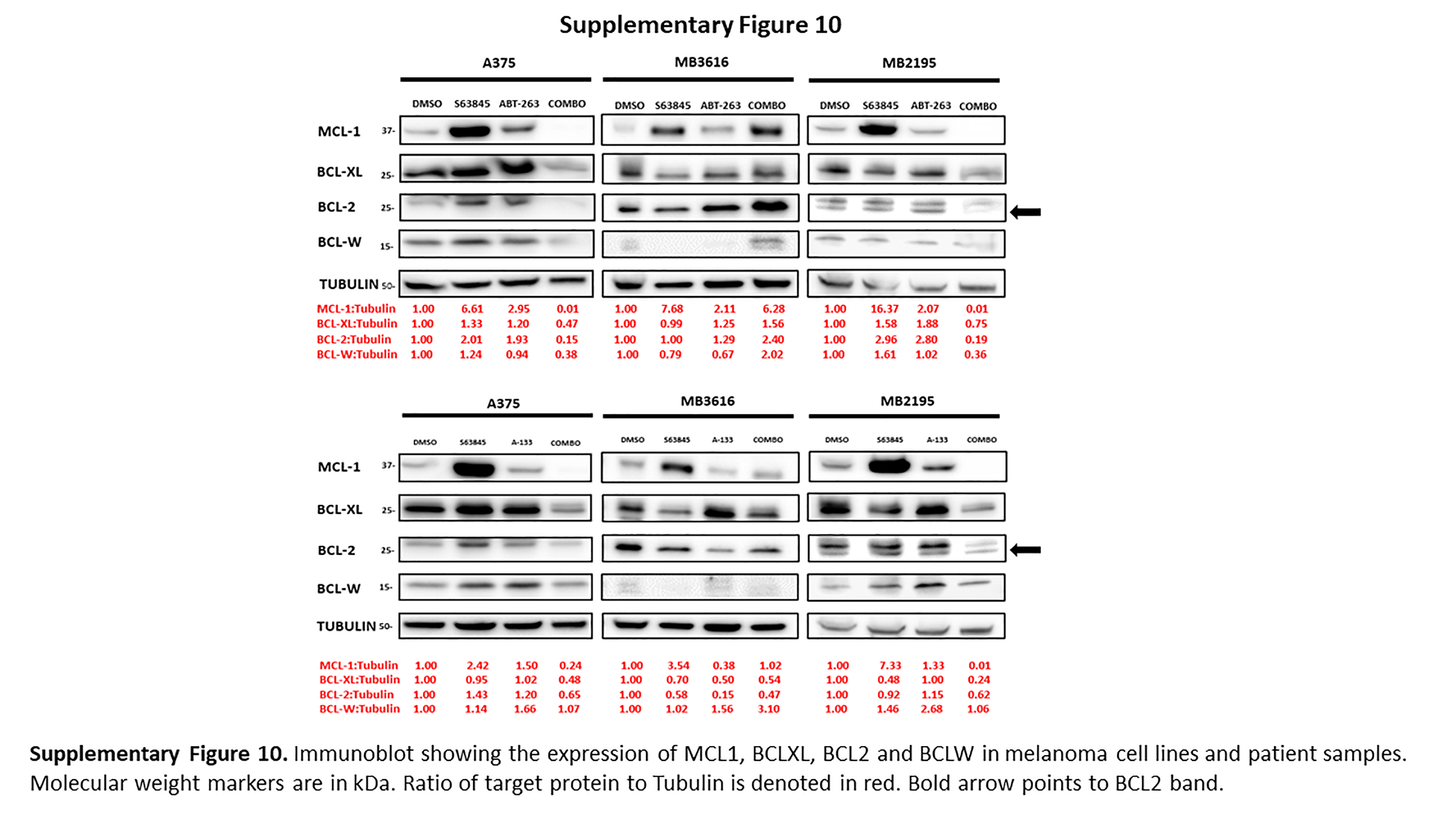

Supplement: Supplementary file 11 — Supplementary Figure 11. [file 41419_2020_2646_MOESM11_ESM.tif]

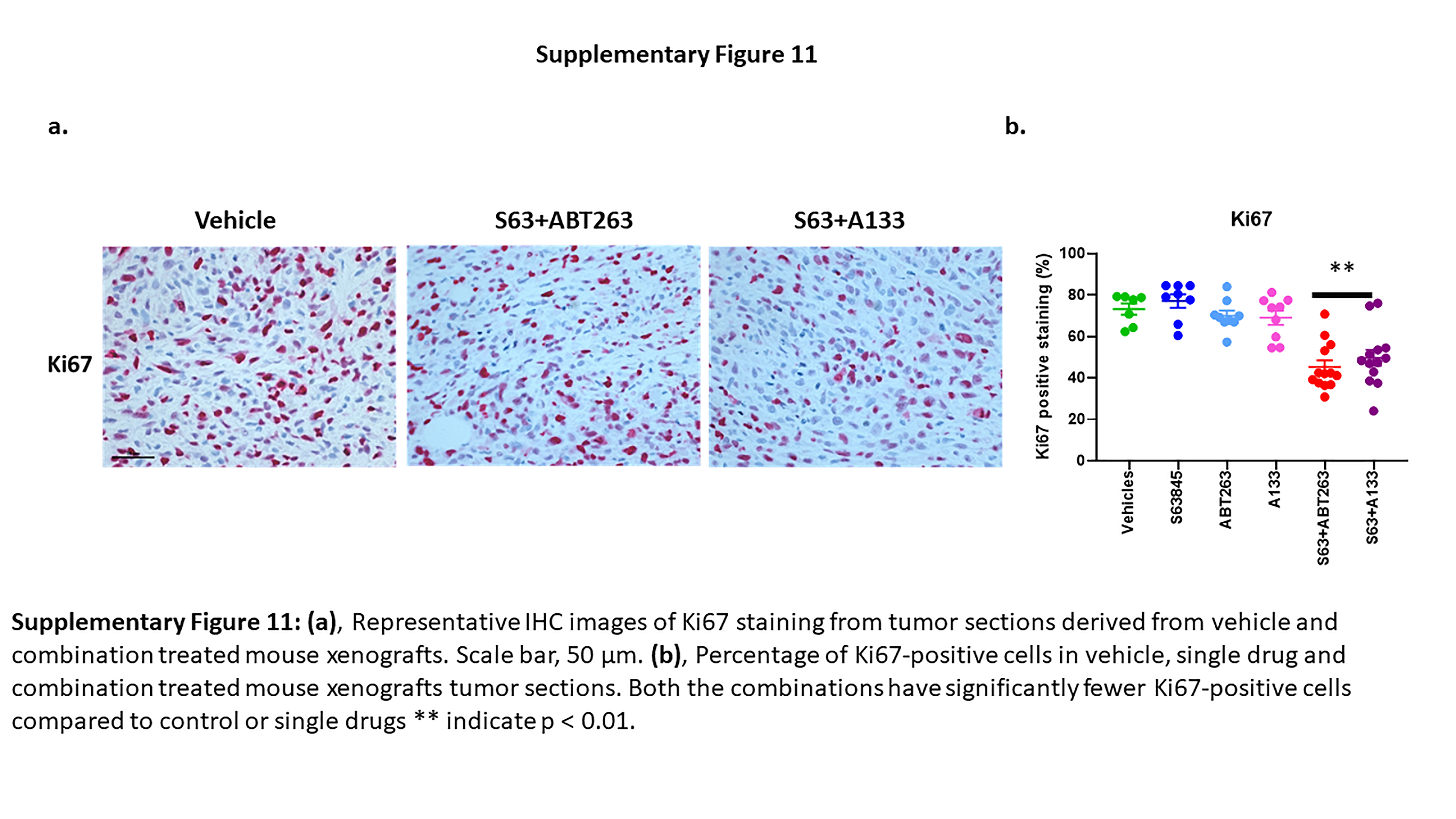

Supplement: Supplementary file 12 — Supplemental Table-1 [file 41419_2020_2646_MOESM12_ESM.tif]
