# Supplementary material for: MCL1 inhibitors S63845/MIK665 plus Navitoclax synergistically kill difficult-to-treat melanoma cells
Source: Cell Death Dis. 2020 Jun 8;11(6):443. doi: 10.1038/s41419-020-2646-2 (PMC7280535; doi:10.1038/s41419-020-2646-2)
Supplement: Supplementary file 13 — Supplemental Table-2 [file 41419_2020_2646_MOESM13_ESM.docx]

**Supplementary Table 1: Details of the melanoma samples used for the study**
