# Supplementary material for: MCL1 inhibitors S63845/MIK665 plus Navitoclax synergistically kill difficult-to-treat melanoma cells
Source: Cell Death Dis. 2020 Jun 8;11(6):443. doi: 10.1038/s41419-020-2646-2 (PMC7280535; doi:10.1038/s41419-020-2646-2)
Supplement: Supplementary file 14 — Supplementary Figure legends-clean copy [file 41419_2020_2646_MOESM14_ESM.docx]

**Supplementary Figure Legends:**

**Supplementary Figure 1. Combination therapy S63845+ABT-263 and S63845+A-1331852, but not ABT-199+A-1331852, are potent to kill melanoma cell lines** (a), (b), (c). ATP assay data of single drug and combination treatments of BH3 mimetics, S63845 plus A-1331852 (a) S63845 plus ABT-263 (b) and ABT-199 plus A-1331852 (c). Y-axis shows percentage of relative viability and X-axis indicates the dosages of drug in µM. Both combination S63845 plus ABT-263 and S63845 plus A-1331852 decreased cell viabilities significantly at sub-micromolar dose (p<0.05 for both combinations). For visual clarity, we have not marked inside the figure. Error bars represent +/- SD.

**Supplementary Figure 2.** Compusyn software (version 1) was used to obtain Combination-Index (CI) of the drug combinations from the data of Figure 1c, Figure 2 and Supplementary Figure 1. CI values < 0.9 (red line) indicate synergism. Smaller CI values indicate stronger synergy^1^.

**Supplementary Figure 3. Combination therapy S63845+ABT-263 and S63845+A-1331852 at 0.156 and 0.625µM doses have modest effect on the normal human melanocytes.** ATP assay data of single drug and combination treatments of BH3 mimetics, (a) S63845 plus ABT-263 (b) S63845 plus A-1331852. Y-axis shows percentage of relative viability and X-axis indicates the dosages of drug in µM. Error bars represent +/- SEM.

**Supplementary Figure 4.** Visual appearance of melanoma cells showing cell death in combination treatments after 48h. These images are representative of the cells collected for immunoblot analysis shown in Figure 2d. Scale bar =100 μm.

**Supplementary Figure 5.** Representative images of A375 and MB3616 cells from IncuCyte live cell analysis with Caspase 3/7 reagent upon indicated treatments for 48 h. The images are representative of the data shown in Figure 3 with single or combination treatment at 625nM. Scale bar =400 μm.

**Supplementary Figure 6.** ATP assay showing combination therapy S63845+ABT-263 and S63845+A-1331852 are potent to kill immunotherapy relapsed melanoma line MB3961. Both combination S63845 plus ABT-263 and S63845 plus A-1331852 decreased cell viabilities significantly at sub-micromolar dose (p<0.001 for both combinations). For visual clarity, we have not marked inside the figure. Error bars represent +/- SEM.

**Supplementary Figure 7. The combinations of S63845 plus A-1331852 or ABT-263 can overcome melanoma’s resistance to current treatments.** (a), Cell lines with acquired resistance to BRAF inhibitor Vemurafenib or MEK inhibitor Trametinib were created on the parental lines A375 and SKMEL-28. The A375 Vem-resistant line and SKMEL-28 Vem resistant lines (red) were quite resistant to 5µM of Vemurafenib compared to their parental lines (green). The SKMEL-28 Tram-resistant line (red) was more resistant to 0.2µM of Trametinib compared to the parental line (green). Y-axis shows percentage of relative viability and X-axis shows doses of drug (μM). (b), ATP assay data of single drug and combination treatments of BH3 mimetics with SKMEL-28 Vemurafenib (VEM) resistant lines. Both combinations decreased cell viabilities significantly at sub-micromolar dose (p<0.01 for both combinations). For visual clarity, we have not marked inside the figure. Error bars represent +/- SD/SEM.

**Supplementary Figure 8.** (a), Representative images from IncuCyte live cell analysis with Caspase 3/7 Reagent with MB2114 upon indicated treatments for 48 h. Scale bar =400 μm. The images are representative of the data shown in Figure 5b. (b), Relative proliferation of MB2114 cells upon DMSO, single drug or combination treatment calculated from IncuCyte live cell imaging. Both the combinations (S63845+ABT263 and S63845 + A-1331852) significantly (p ≤ 0.01) reduced proliferation compared with DMSO or with single drug treated conditions at sub-micromolar dose of 625nM. Error bars represent +/- SEM.

**Supplementary Figure 9.** (a), Representative images from IncuCyte live cell analysis with Caspase 3/7 Reagent with MB4667 upon indicated treatments for 48 h. Scale bar =400 μm. (b), Relative proliferation of MB4667 cells upon DMSO, single drug or combination treatment calculated from IncuCyte live cell imaging. Both the combinations (S63845+ABT263 and S63845 + A-1331852) significantly (p ≤ 0.001) reduced proliferation compared with DMSO or with single drug treated conditions at sub-micromolar dose of 625nM. Error bars represent +/- SEM.

**Supplementary Figure 10.** Immunoblot showing the expression of MCL1, BCLXL, BCL2 and BCLW in melanoma cell lines and patient samples. Molecular weight markers are in kDa. Ratio of target protein to Tubulin is denoted in red. Bold arrow points to BCL2 band.

**Supplementary Figure 11:** (a), Representative IHC images of Ki67 staining from tumor sections derived from vehicle and combination treated mouse xenografts. Scale bar, 50 μm. (b), Percentage of Ki67-positive cells in vehicle, single drug and combination treated mouse xenografts tumor sections. Both the combinations have significantly fewer Ki67-positive cells compared to control or single drugs ** indicate p < 0.01.

References:

1 Chou, T. C. Theoretical basis, experimental design, and computerized simulation of synergism and antagonism in drug combination studies. *Pharmacol Rev* **58**, 621-681, doi:10.1124/pr.58.3.10 (2006).

**Supplementary Table Legends:**

**Supplementary Table 1:** Details of the melanoma samples used for the study

**Supplementary Table 2:** IC50 value of the Patient sample lines and cell lines
