# Supplementary material for: MCL1 inhibitors S63845/MIK665 plus Navitoclax synergistically kill difficult-to-treat melanoma cells
Source: Cell Death Dis. 2020 Jun 8;11(6):443. doi: 10.1038/s41419-020-2646-2 (PMC7280535; doi:10.1038/s41419-020-2646-2)
Supplement: Supplementary file 15 — Supplementary materials and methods (clean version) [file 41419_2020_2646_MOESM15_ESM.docx]

**Supplementary Materials and Methods**

**Melanoma cell lines, either long-established conventional lines or newly established patient lines**

The long-established human melanoma cell lines A375, 1205Lu, Hs294T, and SKMEL-28 are with a *BRAF^V600E^* mutation. WM852c is a *NRAS^Q61R^* mutation. Hs852T were obtained from ATCC (Manassas, VA); WM852c was provided by Dr. Meenhard Herlyn. Patient derived cell lines were provided by the University of Colorado Skin Cancer Biorepository (patient consent and specimen usage outlined under COMIRB 05-0309) and validated by melanoma triple cocktail staining. Patient lines were derived from metastases of patients seen at our institution, and include samples derived from patients relapsed from current treatments. Patient lines were STR profiled with >80% match to the patient’s corresponding blood sample. Genetic backgrounds are listed in Supplementary Table 1. All cell lines were maintained in RPMI 1640 medium (Invitrogen, Grand Island, NY) with 10% fetal bovine serum (Gemini Bio-Products, Inc., West Sacramento, CA) and were tested for mycoplasma. Primary melanocytes HEM_N_MP2 were obtained from Life Technologies (Carlsbad, CA). Melanocytes were maintained in Medium 254 with Human Melanocyte Growth Supplement-2 (Life Technologies, Carlsbad, CA). To mimic melanoma culture conditions, 10% FBS was added for drug assays.

**ATP viability assay, primary and secondary sphere assays**

Cell viability was evaluated via Cell Titer-Glo Luminescent cell viability assay (Promega Corp., Madison, WI) according to the manufacturer’s protocol. All sphere assays were completed as described in our previous publications^1-4^ . The experimental schematic for the primary and secondary sphere assays was described previously^2^. All assays were performed in no less than triplicate for each cell line and repeated at least thrice for each line. Drug treatments began 120 h after seeding in the primary sphere assay and 24 h after seeding in the monolayer ATP assay.

**Immunoblot**

Both floating and adherent cells were collected and lysed using 2x Laemmli buffer (Bio-Rad, Hercules, CA). Samples were analyzed using the immunoblot analysis protocol as described in^5,6^. Immunoblot images presented in this manuscript are from a representative experiment carried out in triplicate. The manufacturers’ suggested dilutions were used for the following antibodies: (Cell Signaling Technologies, Danvers, MA) PARP (#9532), BCL2 (#15071), BCLXL (#2764), BCLW (#2724), BID (#2002), BIM (#2933) , and α/β TUBULIN (#2148); NOXA (# OP180, Millipore Sigma St. Louis, MO); MCL1 (#sc-819, Santa Cruz Biotechnology, Dallas, TX); and HRP-conjugated goat anti-mouse and anti-rabbit antibodies (Cell Signaling Technology, Danvers, MA).

**Creation of short hairpin RNA transduced cell lines and CRISPR/Cas9-mediated BIM knockout cell lines**

ShRNA lentiviral particles (Santa Cruz Biotechnology, Dallas, TX) were used to construct stable cell lines as previously described^5^. BIM-knockout lines were generated using CRISPR/Cas9 technology, as previously described^2,3,7^.

**References:**

1 Mukherjee, N. *et al.* Combining a BCL2 inhibitor with the retinoid derivative fenretinide targets melanoma cells including melanoma initiating cells. *J Invest Dermatol* **135**, 842-850, doi:10.1038/jid.2014.464 (2015).

2 Mukherjee, N. *et al.* Combining a GSI and BCL-2 inhibitor to overcome melanoma's resistance to current treatments. *Oncotarget* **7**, 84594-84607, doi:10.18632/oncotarget.13141 (2016).

3 Mukherjee, N. *et al.* Use of a MCL-1 inhibitor alone to de-bulk melanoma and in combination to kill melanoma initiating cells. *Oncotarget* **8**, 46801-46817, doi:10.18632/oncotarget.8695 (2017).

4 Mukherjee, N. *et al.* Use of a MCL-1 inhibitor alone to de-bulk melanoma and in combination to kill melanoma initiating cells. *Oncotarget* **8**, 46801-46817, doi:10.18632/oncotarget.8695 (2017).

5 Reuland, S. N. *et al.* The combination of BH3-mimetic ABT-737 with the alkylating agent temozolomide induces strong synergistic killing of melanoma cells independent of p53. *PLoS One* **6**, e24294 (2011).

6 Ruth, M. C. *et al.* RhoC promotes human melanoma invasion in a PI3K/Akt-dependent pathway. *J Invest Dermatol* **126**, 862-868 (2006).

7 Mukherjee, N. *et al.* BH3 mimetics induce apoptosis independent of DRP-1 in melanoma. *Cell death & disease* **9**, 907, doi:10.1038/s41419-018-0932-z (2018).
